# Glucose starvation induces fumarate hydratase succinylation and inhibits ferroptosis to maintain colon cancer cell proliferation

**DOI:** 10.1038/s41419-026-08975-9

**Published:** 2026-06-22

**Authors:** L. T. Sun, X. H. Qin, Y. D. Zheng, Z. Y. Zhang, Q. Wang

**Affiliations:** Department of Oncology, The Third Xiangya Hospital, Central South University, Changsha, China

**Keywords:** Cancer metabolism, Post-translational modifications

## Abstract

Cancer cells frequently reside in a glucose-deprived microenvironment due to rapid tumor proliferation and insufficient angiogenesis. However, the mechanisms by which colorectal cancer cells (CRC) adapt to glucose starvation to sustain proliferation remain unclear. Succinylation, a novel post-translational modification, has been implicated in regulating tumor cell proliferation and survival under nutrient stress. Our study reveals that fumarate hydratase (FH), a key enzyme in the tricarboxylic acid (TCA) cycle, is downregulated in CRC and acts as a tumor suppressor. Under glucose starvation mimicked in vitro, FH protein expression is reduced, leading to abnormal accumulation of its upstream metabolites fumarate and succinate, which correlates with advanced clinical stage and poor prognosis in CRC patients. Mechanistically, accumulated fumarate specifically binds to and stabilizes the NRF2 protein, upregulating the expression of GPX4 and FTH1 to inhibit ferroptosis, thereby sustaining CRC cell proliferation. Meanwhile, glucose starvation induces CPT1A-mediated succinylation of FH at residues K66/K80, reducing FH protein stability and promoting its degradation via the autophagy-lysosome pathway. Our findings reveal the critical role of FH and its succinylation in CRC cell adaptation to glucose starvation, inhibiting ferroptosis, and maintaining cancer cell proliferation, providing novel potential targets and a theoretical basis for the clinical treatment of CRC.

## Introduction

Colorectal cancer (CRC) ranks as the third most common malignancy worldwide, with its incidence and mortality continuing to rise globally [1]. Although advances in targeted and immunotherapies have improved outcomes for some metastatic colorectal cancer (mCRC) patients, the 5-year overall survival rate for mCRC patients remains below 10% [2], underscoring substantial clinical challenges. Rapid cancer cell proliferation and inadequate angiogenesis often lead to nutrient deprivation (particularly glucose) within the tumor microenvironment, especially in central regions [3, 4]. Hirayama et al. confirmed that glucose levels in CRC tissues are only one-tenth of those in normal colorectal mucosa [5], indicating that CRC cells persistently reside in a glucose-deprived microenvironment. This presents a metabolic paradox: glucose is an essential substrate for cancer cell energy metabolism and biosynthesis, and its scarcity should theoretically suppress tumor growth. Nevertheless, CRC cells maintain survival and continuous proliferation even under low or no glucose conditions, a capability tightly linked to metabolic reprogramming [6–8]. Ferroptosis, an iron-dependent form of programmed cell death [9], participates in the adaptive response of tumor cells to nutrient-deficient microenvironments by regulating iron metabolism, lipid peroxidation, and redox homeostasis [10–12]. Emerging evidence suggests that ferroptosis may serve as a key regulatory pathway enabling CRC cells to sustain proliferation and survival under glucose starvation; However, the precise mechanisms remain elusive and warrant further investigation. Fumarate hydratase (FH), a critical enzyme in the TCA cycle, catalyzes the conversion of fumarate to malate and participates in cellular energy metabolism and proliferation control [13]. Studies have shown that FH is downregulated in various tumors, including renal cell carcinoma and malignant pheochromocytoma [14–16]. Loss or mutation of FH results in aberrant accumulation of fumarate, which inhibits ferroptosis via the modulation of GSH/GPX4, NRF2/HO-1, and iron metabolism pathways, thereby promoting cancer cell proliferation [17–21]. Under glucose starvation, cancer cells acquire ferroptosis resistance through AMPK-dependent suppression of ROS accumulation or the disruption of excessive unsaturated fatty acid biosynthesis [22, 23]. Whether FH and its upstream metabolite fumarate serve as critical mediators linking glucose deficiency to ferroptosis in CRC adaptation and proliferation remains an unanswered and compelling question. Protein succinylation is an emerging post-translational modification (PTM) that primarily utilizes succinyl-CoA as the donor group, covalently attaching succinyl moieties to lysine residues of substrate proteins through enzymatic or non-enzymatic mechanisms. Compared to other lysine modifications, succinylation introduces a larger structural group and reverses the lysine charge from +1 to −1, often leading to more pronounced alterations in protein structure and function [24]. Accumulating evidence indicates that cancer cells undergoing nutrient stress exhibit aberrant succinylation, which modulates metabolic enzymes and facilitates metabolic reprogramming to sustain rapid proliferation [25–27]. Our preliminary succinyl-proteomic profiling revealed significantly elevated FH succinylation under glucose starvation. Accordingly, this study aims to elucidate the role and regulatory mechanisms of FH and its succinylation in CRC cell adaptation to glucose starvation and maintenance of proliferation, and to delineate the CPT1A-mediated FH succinylation network, thereby identifying novel potential targets for the clinical treatment of CRC.

## Materials and methods

### Bioinformatics databases

The colon cancer datasets GSE41258, GSE87211, GSE39582, and GSE17538 were obtained from the Gene Expression Omnibus (GEO). TCGA-COAD and TCGA-READ (colorectal cancer) datasets were downloaded from UCSC Xena (University of California, Santa Cruz, CA, USA). Differential expression analysis was performed using the limma package in R software (version 4.4.0; R Foundation for Statistical Computing, Vienna, Austria). Survival analyses were performed using the survival package. Kaplan–Meier curves were compared using the log-rank test. Cox proportional hazards regression models were used to evaluate the independent prognostic value of FH, and hazard ratios (HR) with 95% confidence intervals (CI) were reported.

### Patients and tissue specimens

From February 2023 to February 2024, fresh surgical specimens from 68 CRC patients and matched adjacent non-tumor tissues were collected at The Third Xiangya Hospital of Central South University (Changsha, China). The samples were rinsed with saline, immediately frozen in dry ice, and stored at −80 °C. This study was conducted in accordance with the principles of the Declaration of Helsinki, and approved by the Ethics Committee of The Third Xiangya Hospital of Central South University (Ethics approval number: 2025-S738), and written informed consent was obtained from all patients.

### Cell culture and treatments

The CRC cell lines HCT116, Colo205, LS174T, T84, and RKO were purchased from Abiowell Biotechnology (Abiowell Biotechnology Co., Ltd., Changsha, China), while HT29, LS180, and Caco-2 were obtained from Yihe Biotechnology Technology (Yihe Biotechnology Co., Ltd., Shanghai, China). All cell lines were identified by short tandem repeat (STR) profiling and tested mycoplasma-free. Different cells were cultured in corresponding media supplemented with 10% fetal bovine serum (FBS; Gibco, Thermo Fisher Scientific, Waltham, MA, USA) and 1% streptomycin/penicillin: HCT116 and HT29 cells in McCoy’s 5 A medium; Colo205 and Caco-2 cells in RPMI-1640 medium; T84 cells in DMEM/F12 medium; LS174T cells in MEM medium with non-essential amino acids (NEAA); RKO and LS180 cells in MEM medium without NEAA. Cultures were performed at 37 °C with 5% CO_2_. For glucose starvation experiments, HCT116 and LS174T cells were cultured in medium until 80% confluency, then switched to media containing 5 mmol/L glucose (normal glucose, Glu(+)), 10 mmol/L glucose (low glucose), or 0 mmol/L glucose (glucose starvation, Glu(–)).

### Quantitative reverse transcription PCR (qRT-PCR)

Total RNA was isolated from cells and tissues using the SteadyPure Quick RNA Extraction Kit (Accurate Biotechnology Co., Ltd., Changsha, China). cDNA synthesis was performed with the Evo M-MLV RT Premix Kit (Accurate Biology, China). qRT-PCR utilized the SYBR® Green Pro Taq HS Premix Kit (Accurate Biology, China). Analyses were performed on a LightCycler® 96 Real-Time PCR System (Roche Diagnostics, Basel, Switzerland), and relative gene expression was calculated using the 2^−ΔΔCt^ method. Gene expression was normalized to β-actin. The primer sequences are listed in Supplementary Table 1.

### Western blot

Total protein was extracted using the Total Protein Extraction Kit (Beyotime Biotechnology, Shanghai, China). The lysates were centrifuged at 10,000 rpm for 10 min at 4 °C. Proteins were separated by SDS-PAGE and transferred to nitrocellulose membranes (Pierce, USA). Membranes were blocked with 5% non-fat milk for 1 h at room temperature, incubated with primary antibodies overnight at 4 °C, followed by secondary antibody incubation for 1 h at room temperature. Membranes were treated with ECL substrate (Beyotime Biotechnology, China) and imaged with a digital gel imaging system (Tanon, 5500 Multi, Shanghai, China). The antibodies are detailed in Supplementary Table 2.

### Plasmid construction and cell transfection

All knockdown and overexpression plasmids, as well as wild-type (FH-WT) and point-mutated (FH-K66R, FH-K80R) plasmids, were designed and synthesized by Shanghai Gemma Co., Ltd. Cells were seeded into 6-well plates for 24 h, followed by transfection with siRNAs and plasmids using Lipofectamine® 3000 (Invitrogen, Thermo Fisher Scientific, Carlsbad, CA, USA). The transfected cells were cultured for 6–12 h. To ensure transfection efficiency, multiple siRNA sequences were applied for some genes. The siRNA and shRNA sequences are listed in Supplementary Table 3.

### Immunohistochemistry (IHC)

The nude mice tumor tissues or surgically resected specimens were fixed and embedded in paraffin for sectioning. Sections were dewaxed in xylene I, II (Sinopharm Chemical Reagent Co., Ltd., Shanghai, China) and ethanol gradients. Endogenous peroxidase activity was blocked with 3% H2O2 for 10 min, followed by blocking with non-immune serum for 20 min at room temperature. Sections were incubated overnight at 4 °C with primary antibodies against FH (1:1000; Proteintech Group, Rosemont, IL, USA) or Ki-67 (1:200; Proteintech, USA), then with HRP-conjugated secondary antibodies for 30 min at room temperature. Signals were developed with DAB substrate (Zhongshan Golden Bridge Biotechnology Co., Ltd., Beijing, China) for approximately 2 min, and stopped with distilled water. Counterstaining was performed with hematoxylin for 30 s. Finally, sections were dehydrated through ethanol, cleared in xylene, and mounted with neutral gum.

### CCK8 cell proliferation assay

HCT116 and LS174T cells were seeded in 96-well plates and cultured in complete medium. CCK8 solution (10 μL; Beyotime Biotechnology, China) was added to each well, marking the 0-h time point. The cells were incubated for specified durations, and optical density (OD) at 450 nm was measured using a microplate reader (Bio-Rad Laboratories, Hercules, CA, USA). Cell proliferation rates were calculated based on the OD values.

### Transwell cell invasion assay

HCT116 and LS174T cells were serum-starved for 24 h. Matrigel matrix (Corning Incorporated, Corning, NY, USA) was diluted 1:8 on ice, added to Transwell inserts (Corning, USA), placed in a 4 °C for 15 min, and then incubated at 37 °C for 2 h. Subsequently, suspension (200 μL) was added to the upper chamber, and serum-containing medium (500 μL) was added to the lower chamber. After culturing in an incubator for 24–48 h, the inserts were removed, rinsed with phosphate-buffered saline (PBS), fixed with 4% paraformaldehyde for 20 min, and stained with 0.1% crystal violet for 10–15 min. Invasive cells were counted under an inverted microscope using ImageJ.

### Detection of ferrous ions (Fe^2+^)

HCT116 and LS174T cells were seeded into fluorescence culture dishes (Jet Biofil, Guangzhou, China) and incubated overnight. The cells were treated with specific reagents or transfected with plasmids, and further cultured for 24–48 h. The supernatant was discarded, and the cells were washed 2–3 times with serum-free medium and incubated with 1 μmol/L FerroOrange working solution (Dojindo Molecular Technologies, Kumamoto, Japan) for 30 min at 37 °C. The intracellular Fe^2+^ levels were assessed and imaged by a fluorescence microscope (Axiovert 200, Carl Zeiss, Germany).

### Detection of reactive oxygen species (ROS)

The cells were collected and incubated overnight at 37 °C. C11 BODIPY 581/591 stock solution (2 µL of 10 mmol/L; Thermo Fisher Scientific, USA) was added to the medium and incubated for 30 min at 37 °C under 5% CO_2_. Subsequently, the cells were centrifuged at 1200 rpm for 5 min, the supernatant was discarded, and the cells were resuspended in PBS. The lipid ROS content in cells was detected by flow cytometry (FC 500, Beckman Coulter), and the fluorescence intensity was analyzed by ImageJ.

### Immunofluorescence

The cells were incubated with Lyso-Tracker Green staining working solution (Beyotime Biotechnology, China) for 20–30 min at 37 °C. After incubation with primary antibodies for 2 h, then 50 μL offluorophore-conjugated secondary antibodies were added for 40 min. The nuclei were stained with DAPI (Beyotime Biotechnology, China), and slides were mounted with water-soluble mounting medium (Southern Biotech, USA). Fluorescence microscopy was performed using a Zeiss LSM 710 confocal microscope (Zeiss Microscopy, Germany). Colocalization of target proteins with lysosomes was analyzed using ImageJ.

### Mass spectrometry analysis

HCT116 cells were cultured under glucose starvation and normal glucose conditions. Lysine succinylation modifications and specific lysine sites were analyzed by liquid chromatography-tandem mass spectrometry (LC-MS/MS). This detection process was completed by Jingjie Biotechnology (Jingjie Biotechnology Co., Ltd., Hangzhou, China).

### Co-immunoprecipitation (Co-IP)

HCT116 and LS174T cells were lysed in pre-cooled lysis buffer on ice for 30 min. The protein lysate was incubated with Protein A + G Agarose beads (20 μL; Beyotime Biotechnology, China) at 4 °C for 2 h. After centrifugation, part of the supernatant was reserved as Input. The remaining supernatant was subjected to Co-IP by incubation with specific IP antibodies or control IgG overnight at 4 °C. The beads were washed extensively, and bound proteins were eluted in 1x SDS-PAGE loading buffer (Beyotime Biotechnology, China) by boiling for 3–5 min. IP and Input samples were analyzed by western blotting to assess succinylation levels and target protein expression. The antibodies are listed in Supplementary Table 4.

### Detection of fumarate and succinate levels

Intracellular fumarate and succinate levels were measured, respectively, using the Fumarate Assay Kit and Succinate Assay Kit (Abcam plc, Cambridge, UK). HCT116 and LS174T cells were homogenized in Assay Buffer and centrifuged to remove insoluble debris. The supernatants were transferred to a 96-well plate with standard dilutions for the calibration curve. Then, the reaction mixture (50 μL) was added to each well, incubated at 37 °C in the dark. The absorbance at 450 nm was measured using a microplate reader (Bio-Rad, USA), and metabolite concentrations were calculated based on the standard curve.

### Animal experiments

All animal procedures were approved by the Animal Center of Central South University in accordance with international guidelines and protocols. BALB/c nude mice (Hunan SJA Laboratory Animal Co., Ltd., China) were housed in a specific pathogen-free laboratory. HCT116 cells were subcutaneously injected into the dorsal flank to establish xenograft models. For the FH–Fer-1 experiment, mice bearing FH-overexpressing or control tumors were randomized after tumor establishment, and FH-overexpressing mice received either Fer-1 or saline control by local administration. For metabolic intervention experiments, tumor-bearing mice were treated with dimethyl fumarate (DMF) or diethyl succinate (DES) at indicated doses, while control groups received vehicle. Tumor size was measured using calipers at regular intervals, and tumor volume was calculated. At the experimental endpoint, mice were euthanized, and tumor tissues were harvested for subsequent pathological examination and immunohistochemical analyses.

### In vitro succinylation assay

Purified recombinant CPT1A and Flag-FH (WT) or Flag-FH (K66R/K80R) proteins were incubated in succinylation buffer supplemented with succinyl-CoA (1 mM) at 37 °C. Reactions were terminated by adding SDS loading buffer and boiling at 95 °C for 5 min. Proteins were resolved by SDS-PAGE and analyzed by western blotting using a pan-anti-succinyllysine antibody to detect FH succinylation at the molecular weight corresponding to FH. FH protein input was verified by immunoblotting with anti-FH or anti-Flag antibodies.

### Transmission electron microscopy (TEM)

Cells were collected into 1.5-mL microcentrifuge tubes, washed twice with PBS, and pelleted by low-speed centrifugation (≈300×*g*, 5–8 min). Cell pellets were gently fixed with pre-cooled 2.5% glutaraldehyde at 4 °C for 4–6 h without resuspension. Samples were then post-fixed with 1% osmium tetroxide, dehydrated through a graded ethanol series, embedded in resin, and sectioned into ultrathin slices. Sections were stained with uranyl acetate and lead citrate and examined by transmission electron microscopy (JEM-1400, JEOL Ltd., Tokyo, Japan). Mitochondrial ultrastructural features characteristic of ferroptosis were assessed.

### ELISA measurement of intracellular 4-HNE and MDA

Intracellular lipid peroxidation products, including 4-hydroxynonenal (4-HNE) and malondialdehyde (MDA), were measured using commercial ELISA kits according to the manufacturers’ protocols. Cells were collected after the indicated treatments, washed with ice-cold PBS, and lysed in the supplied lysis buffer containing protease inhibitors. After centrifugation (12,000×*g*, 10–15 min, 4 °C), supernatants were subjected to ELISA analysis. Protein concentrations were determined by BCA assay, and 4-HNE and MDA levels were normalized to total protein content. Absorbance was recorded using a microplate reader, and concentrations were calculated based on standard curves.

### Fumarate hydratase (FH) enzymatic activity assay

Fumarate hydratase (FH) activity was measured using a commercial FH Activity Assay Kit (Beyotime Biotechnology, China; Cat. No. S0520S) according to the manufacturer’s instructions. Cells were harvested after the indicated treatments and lysed in the provided buffer. After centrifugation (12,000×*g*, 10 min, 4 °C), supernatants were collected and protein concentrations were determined by BCA assay. FH activity was monitored by a coupled colorimetric reaction. Equal amounts of protein were incubated with the reaction mixture at 37 °C, and absorbance at 340 nm was recorded using a microplate reader. Enzymatic activity was calculated from the change in absorbance and normalized to total protein content. All experiments were performed in triplicate.

### Molecular docking

Molecular docking was performed to assess the potential interaction between NRF2 and fumarate, following published protocols (PMID: 38639442; 38237597; 40325218). The predicted human NRF2 structure (UniProt Q16236) was obtained from the AlphaFold database, and the fumarate structure (PubChem CID: 5460307) was retrieved from PubChem. Protein preparation and docking were conducted using PyMOL and AutoDock Vina (v1.5.2), with a grid box of 26 × 26 × 26 A covering the predicted binding region. Binding affinity was evaluated based on binding free energy, and the lowest-energy pose (_6.3 kcal/mol) was selected as the optimal conformation. Docking results were visualized using PyMOL.

### Microscale thermophoresis (MST)

Microscale thermophoresis (MST) was performed to assess the binding affinity between fumarate (DMF) and NRF2. GFP-NRF2 wild-type (WT), a binding-site mutant (MT; five residues mutated to alanine), or GFP control were expressed in HEK293T cells, and lysates were collected 48 h post-transfection. DMF was serially diluted (0.005–164 μM) in MST buffer (1 × PBS-P, 5% DMSO) and incubated with GFP-tagged proteins. Measurements were carried out using a Monolith NT.115 (NanoTemper Technologies GmbH, Munich, Germany), and dissociation constants (Kd) were calculated with NanoTemper software.

### Cellular thermal shift assay (CETSA)

Cellular thermal shift assays (CETSA) were performed to assess the thermal stability of NRF2 upon fumarate treatment. HCT116 cells (1 × 10^6^ cells per group) were treated with DMSO, fumarate (10 mM), or ML385 (5 μM, positive control) for 24 h. Cells were then harvested, washed, and resuspended in PBS. Cell suspensions were aliquoted and heated for 3 min at the indicated temperatures (37, 42, 47, 52, 57, 62, and 67 °C), followed by three freeze–thaw cycles at 4 °C. Samples were centrifuged at 12,000×*g* for 10 min to collect the soluble fractions. NRF2 protein levels were analyzed by western blotting, with β-actin used as a loading control.

### Statistical analysis

All experiments were performed in at least three independent biological replicates. Data distribution was assessed using the Shapiro–Wilk normality test. For normally distributed data, results are presented as mean (standard deviation, SD). For non-normally distributed data, results are expressed as median (interquartile range). Comparisons between two groups were performed using unpaired two-tailed Student’s *t*-test or Mann–Whitney U test as appropriate. Comparisons among multiple groups were analyzed using one-way or two-way analysis of variance (ANOVA) followed by Tukey’s post hoc test. Survival curves were generated using the Kaplan–Meier method and compared by the log-rank test. Hazard ratios (HR) with 95% confidence intervals (CI) were calculated using Cox proportional hazards regression models. All statistical analyses were conducted using R software (version 4.4.0; R Foundation for Statistical Computing, Vienna, Austria) or GraphPad Prism (version 10.0; GraphPad Software, San Diego, CA, USA). All statistical tests were two-sided, and *p* < 0.05 was considered statistically significant.

## Results

### FH is downregulated in CRC and functions as a tumor suppressor by inhibiting CRC cell proliferation and invasion

**Fig. 1:**
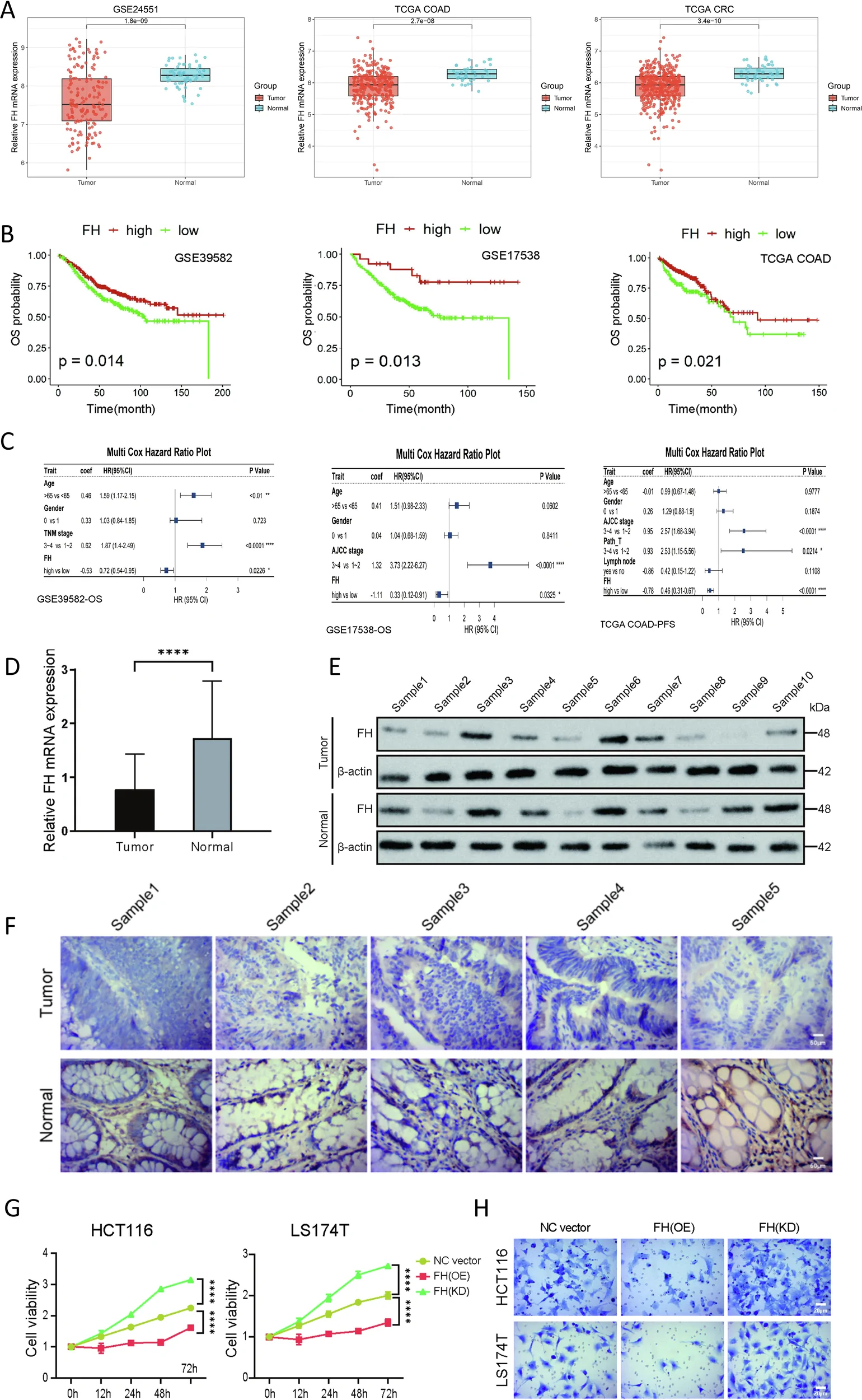
FH is downregulated in CRC and inhibits cell proliferation and invasion. **A** Box plots show the differential expression of FH between colorectal cancer tissues and normal colon tissues across multiple datasets. **B** Kaplan–Meier survival curves demonstrate the prognostic significance of FH expression levels in multiple datasets. **C** Forest plot illustrates the independent prognostic value of FH and other clinical factors in colorectal cancer. **D**–**F** qRT-PCR, western blot, and immunohistochemistry (IHC) were performed to evaluate FH expression in colorectal cancer tissues and matched normal colon tissues (IHC scale bar: 50 μm). **G**, **H** CCK-8 and Transwell assays were used to assess the effects of FH overexpression or knockdown on the proliferation and invasion of HCT116 and LS174T cells (scale bar: 20 μm). Data are presented as the mean ± SD of three independent experiments. ****p < 0.0001.

Analysis of public transcriptomic datasets (GSE24551, TCGA-CRC, and TCGA-COAD) revealed that FH expression was significantly lower in CRC tissues than in adjacent normal tissues. Multi-cohort survival analysis (GSE39582, GSE17538, TCGA-COAD) further demonstrated that high FH expression correlated with longer overall survival (OS) in CRC patients. Multivariate Cox regression analysis identified FH as an independent protective factor in CRC (Fig. 1A–C). To validate these findings, we examined FH expression in 68 paired clinical specimens and confirmed significant downregulation of both FH mRNA and protein in tumor tissues (Fig. 1D–F). Among eight CRC cell lines, HCT116 and LS174T exhibited moderate FH expression and were selected for subsequent functional experiments (Fig. S1A). CCK-8 and Transwell assays demonstrated that FH knockdown significantly enhanced CRC cell proliferation and invasion, whereas FH overexpression exerted the opposite effects, supporting a tumor-suppressive role of FH in CRC (Fig. 1G, H).

### Under glucose starvation, FH protein is degraded via the autophagy-lysosome pathway, impacting CRC cell proliferation

**Fig. 2:**
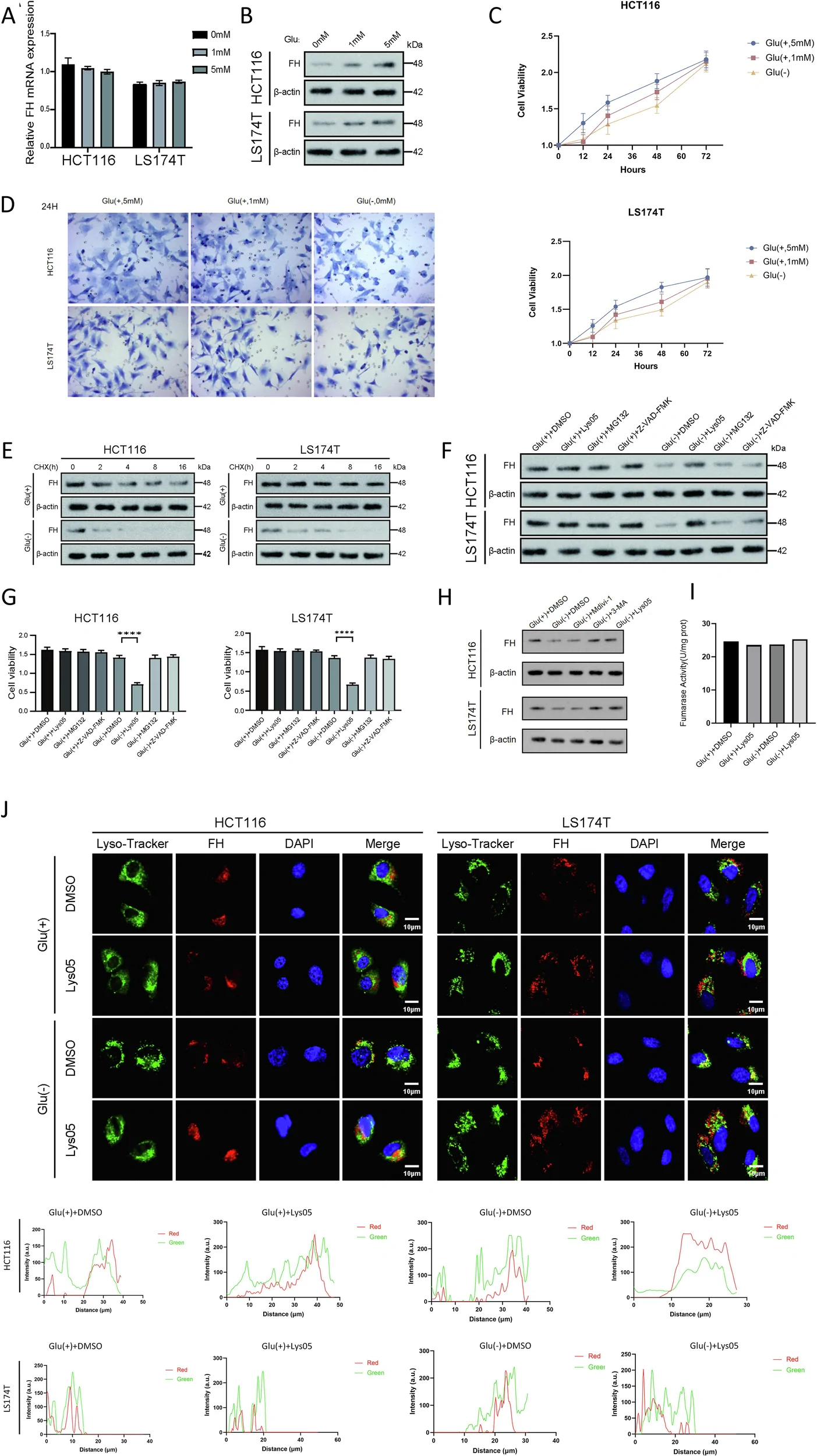
FH protein is degraded via the autophagy-lysosome pathway, impacting CRC cell proliferation. **A**, **B** qRT-PCR and western blot were performed to detect FH mRNA and protein expression in HCT116 and LS174T cells cultured under different glucose concentrations (0 mM, 1 mM, 5 mM). **C**, **D** CCK-8 and Transwell assays were used to evaluate the proliferation and invasion of HCT116 and LS174T cells under different glucose conditions. **E** Western blot was conducted to examine FH protein expression in HCT116 and LS174T cells treated with cycloheximide (CHX) for different time periods (0, 2, 4, 8, 16 h) in the presence or absence of glucose. **F**, **G** Western blot and CCK-8 assays were performed to detect FH protein expression and cell proliferation in HCT116 and LS174T cells after treatment with inhibitors of different protein degradation pathways for 24 h. **H** Under glucose starvation conditions, western blot was used to determine FH protein expression in cells treated with different autophagy inhibitors (mitophagy inhibitor Mdivi-1, general autophagy inhibitor 3-MA, and Lys05). **I** FH-specific activity was measured by the FH enzyme activity assay in the presence or absence of glucose and/or Lys05 treatment. **J** Confocal microscopy was used to observe the immunofluorescent colocalization of FH (red) and lysosomes (green) in HCT116 and LS174T cells in the presence or absence of glucose and/or Lys05 treatment (scale bar: 10 μm). Quantitative analysis of the FH protein and autophagy markers was also performed. *n* = 3 biological replicates per group. Data are presented as mean ± SD. ns, not significant, *p < 0.05, **p < 0.01, ***p < 0.001, ****p < 0.0001).

To mimic the glucose-deprived microenvironment of CRC cells in vitro, we cultured HCT116 and LS174T cells under normal glucose (Glu(+), 5 mmol/L), low glucose (1 mmol/L), and glucose starvation (Glu(−), 0 mmol/L) conditions. qRT-PCR and Western blot analyses showed that FH mRNA levels remained unchanged, whereas FH protein levels decreased dramatically with declining glucose concentrations (Fig. 2A, B). CCK-8 and Transwell assays revealed that cell proliferation was markedly suppressed during the initial phase of glucose starvation, but began to recover after 24 h and approached near-normal levels by 72 h. This indicates that CRC cells gradually adapt to glucose deprivation and restore proliferative capacity. Thus, the 24 h time point was selected for subsequent experiments (Fig. 2C, D). To investigate the molecular basis of glucose starvation-induced FH downregulation, cycloheximide (CHX) chase assays were performed. FH protein degradation was markedly accelerated under Glu(−)conditions, with a significantly shortened half-life, indicating reduced protein stability (Fig. 2E). To identify the degradation pathway, cells were treated with the autophagy-lysosome inhibitor Lys05, the proteasome inhibitor MG132, or the caspase inhibitor Z-VAD-FMK under Glu(−) conditions. Only Lys05 significantly restored FH protein levels, which were accompanied by reduced cell proliferation; MG132 and Z-VAD-FMK had no apparent effects, indicating that FH degradation mainly depends on the autophagy-lysosome pathway (Fig. 2F, G). Further treatment with the mitophagy inhibitor Mdivi-1, the autophagy inhibitor 3-MA, and Lys05 under Glu(−) conditions confirmed that FH degradation occurs via the general autophagy-lysosome pathway rather than mitophagy (Fig. 2H). As a key TCA cycle enzyme, FH-specific enzyme activity assays showed no significant changes in specific activity regardless of glucose availability or Lys05 treatment, indicating that glucose starvation modulates FH protein abundance rather than its catalytic function (Fig. 2I). Confocal microscopy demonstrated enhanced colocalization of FH with lysosomes under Glu(−) conditions, which was markedly attenuated by Lys05. This further supports the conclusion that FH is degraded via the autophagy-lysosome pathway (Fig. 2J).

### Glucose starvation induces FH downregulation, which sustains CRC cell proliferation and survival by inhibiting ferroptosis

**Fig. 3:**
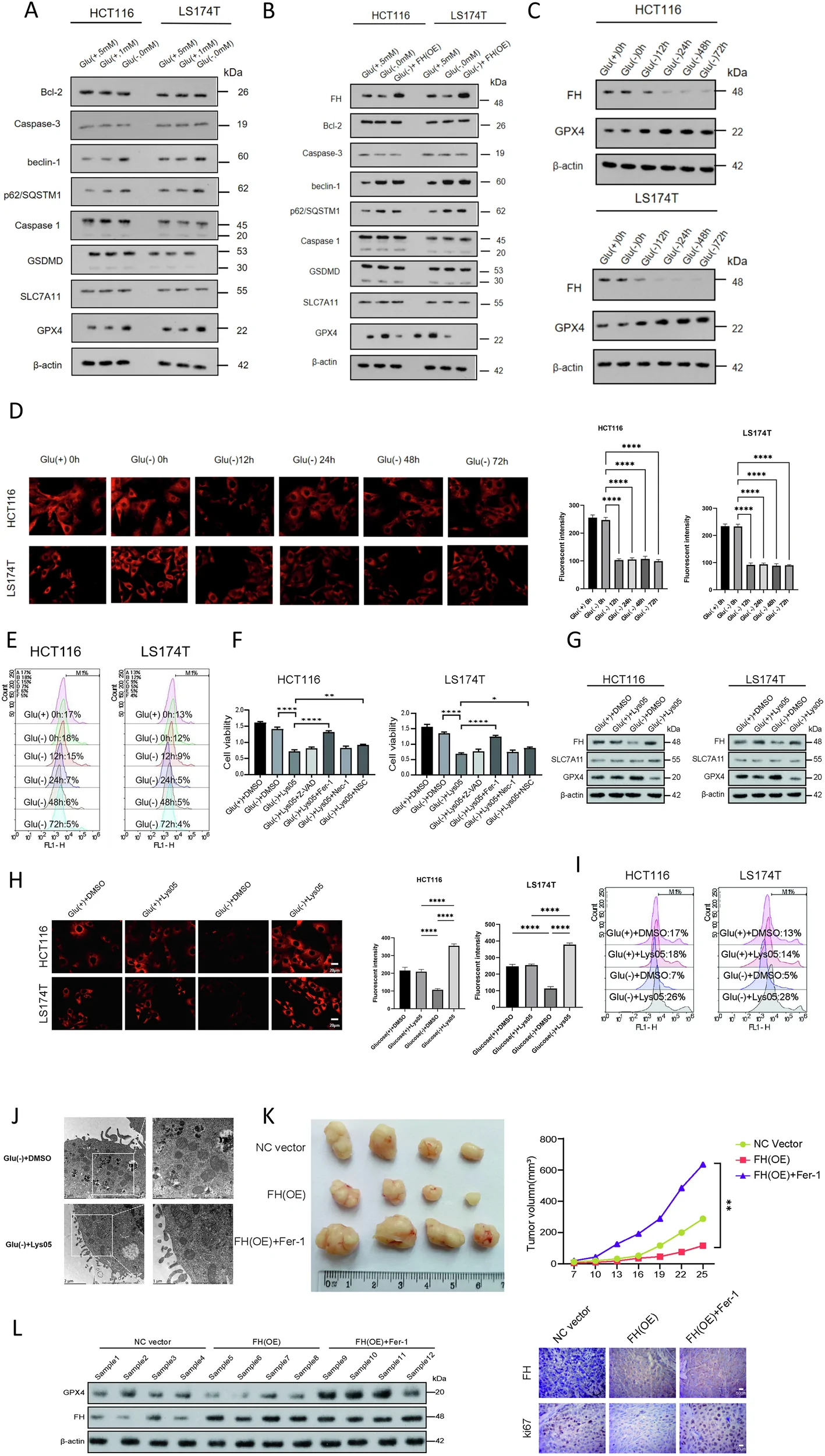
Glucose starvation induces FH downregulation, which sustains CRC cell proliferation by inhibiting ferroptosis. **A** Western blot was performed to detect the expression of apoptosis (Bcl-2, cleaved 743 caspase-3), autophagy (beclin-1, p62), pyroptosis (Caspase-1, GSDMD), and ferroptosis markers (GPX4, SLC7A11) in HCT116 and LS174T cells under different glucose concentrations (0 mM, 1 mM, 5 mM). **B** Western blot was used to examine the expression of apoptosis, autophagy, pyroptosis, and ferroptosis markers in HCT116 and LS174T cells with FH overexpression under glucose starvation conditions. **C** Western blot was conducted to determine the protein expression of FH and GPX4 in HCT116 and LS174T cells at different time points (0, 12, 24, 48, 72 h) under glucose starvation conditions. **D** FerroOrange fluorescent probe was used to perform immunofluorescent staining and quantitative analysis of intracellular Fe²⁺ levels in HCT116 and LS174T cells at different time points under glucose starvation conditions (scale bar: 20 μm). **E** Flow cytometry was employed to detect lipid ROS levels using C11 BODIPY 581/591 staining in HCT116 and LS174T cells at different time points under glucose starvation conditions. **F** CCK-8 assay was performed to evaluate the proliferation of HCT116 and LS174T cells treated with various cell death inhibitors under glucose starvation conditions. **G** Western blot was used to detect the protein expression levels of GPX4 and SLC7A11 in HCT116 and LS174T cells treated with or without glucose or Lys05. **H** FerroOrange fluorescent probe was employed to detect intracellular Fe²⁺ levels in HCT116 and LS174T cells treated with or without glucose or Lys05. **I** Flow cytometry was performed to determine lipid ROS levels using C11 BODIPY 581/591 staining in HCT116 and LS174T cells. **J** Transmission electron microscopy (TEM) showed the mitochondrial ultrastructure of HCT116 cells under glucose starvation and after FH expression restoration by Lys05 treatment (scale bar: 2 μm). **K** Tumor growth in xenograft models of the NC vector group, FH overexpression group, and FH(OE) + Fer-1 group. Representative images of tumors and curves showing tumor volume changes over time are presented. **L** Western blot was used to detect the expression of FH and GPX4 in xenograft tumor tissues, and IHC was performed to examine Ki-67 staining in tumor tissues (scale bar: 50 μm). *n* = 3 biological replicates per group. Data are presented as mean ± SD. ns, not significant, *p < 0.05, **p < 0.01, ***p < 0.001, ****p < 0.0001.

Previous studies have shown that glucose starvation confers ferroptosis resistance in tumor cells, and fumarate accumulation caused by FH inactivation is also involved in ferroptosis regulation. In HCT116 and LS174T cells subjected to a gradient of glucose concentrations, we found that the Glu(−) group exhibited upregulated expression of the autophagy-related proteins beclin-1 and p62, as well as the ferroptosis-related protein GPX4, with no change in SLC7A11. No significant changes were detected in apoptosis (Bcl-2, cleaved caspase-3) or pyroptosis (Caspase-1, GSDMD) markers (Fig. 3A). To assess whether these alterations are FH-dependent, FH was overexpressed under glucose starvation, resulting in markedly decreased GPX4 expression without obvious changes in beclin-1 or p62, suggesting that restored FH promotes ferroptosis without affecting autophagy (Fig. 3B). Kinetic analysis of ferroptosis under glucose starvation (0, 12, 24, 48, 72 h) showed that ferroptosis was inhibited in the early phase, with maximal suppression observed at 24 h, which persisted until 72 h. This was evidenced by upregulated GPX4 and reduced intracellular Fe²⁺ and lipid ROS levels (Fig.3C–E). This pattern closely matched the adaptive recovery of cell proliferation after 24 h of glucose deprivation.

To determine whether additional cell death modalities contribute to CRC proliferation under glucose starvation, FH expression was restored using Lys05, and cells were treated with various cell death inhibitors. Only the ferroptosis inhibitor Ferrostatin-1 (Fer-1) significantly reversed the proliferation inhibition, whereas the apoptosis inhibitor Z-VAD, the necroptosis inhibitor Nec-1, and the pyroptosis inhibitor NSC had negligible effects (Fig. 3F). Further validation using Fer-1 and the iron chelator deferoxamine (DFO) showed reduced lipid peroxidation products (4-HNE and MDA) and restored cell viability, indicating that FH modulates CRC cell proliferation via ferroptosis (Fig. S1B). Restoration of FH under Glu(−) decreased GPX4 expression and increased intracellular Fe²⁺ and lipid ROS levels (Fig. 3G–I). Transmission electron microscopy (TEM) revealed typical ferroptotic mitochondrial ultrastructural alterations, including shrunken mitochondria, increased membrane density, and disrupted mitochondrial cristae (Fig. 3J). In vivo, FH overexpression suppressed tumor growth in xenograft models, an effect partially reversed by Fer-1 administration (Fig. 3K). Western blot and immunohistochemistry (IHC) confirmed downregulated GPX4 and low Ki-67 expression in FH-overexpressing tumors, whereas Fer-1 treatment inhibited ferroptosis and restored proliferation (Fig. 3L). Collectively, these findings demonstrate that reduced FH under glucose starvation sustains CRC cell proliferation and survival by inhibiting ferroptosis.

### Abnormal accumulation of fumarate and succinate inhibits ferroptosis and maintains CRC cell proliferation

**Fig. 4:**
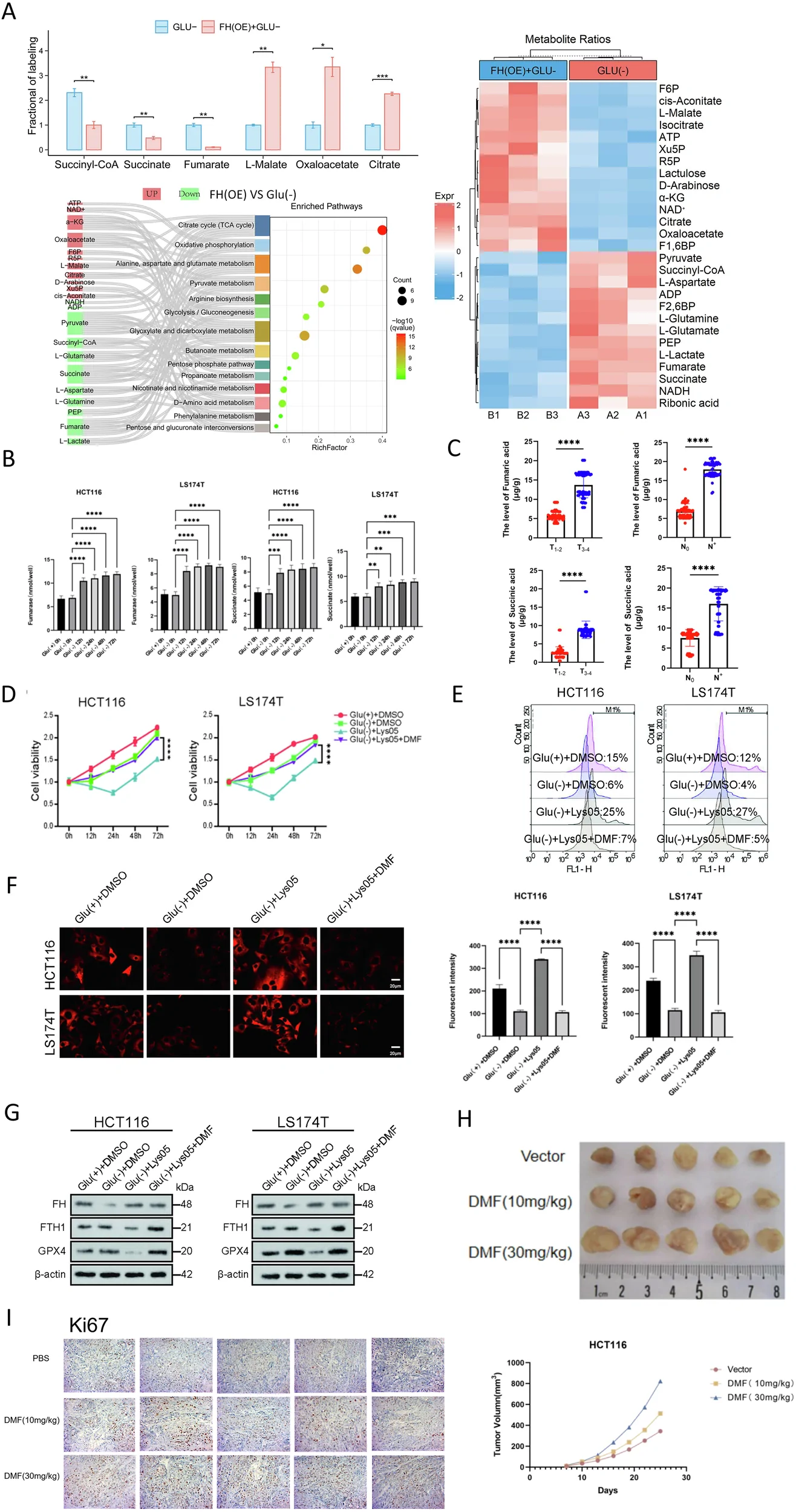
Abnormal accumulation of fumarate and succinate inhibits ferroptosis and maintains CRC cell proliferation. **A** Metabolomic profiling was performed to detect metabolomic changes between the Glu(−) group and the Glu(−) + FH(OE) group. Left: Bar graphs show the changes in key metabolites (succinyl-CoA, succinate, fumarate, L-malate, oxaloacetate, citrate) under different conditions. Right: Heatmap shows the changes in the ratios of various metabolites. Data are from *n* = 3 independent biological replicates. **B** Enzymatic colorimetric assay was used to detect the accumulation of fumarate and succinate in HCT116 and LS174T cells at different time points (0, 12, 24, 48, 72 h) under glucose starvation conditions. **C** Detection of fumarate and succinate levels in serum samples from 68 clinical CRC patients. **D** CCK-8 assay was performed to detect the proliferative capacity of HCT116 and LS174T cells after DMF treatment. **E** Flow cytometry was employed to detect lipid ROS levels using C11 BODIPY 581/591 staining in HCT116 and LS174T cells after DMF treatment. **F** FerroOrange fluorescent probe was used to perform immunofluorescent staining and quantitative analysis of intracellular Fe²⁺ levels in HCT116 and LS174T cells after DMF treatment (scale bar: 20 μm). **G** Western blot was performed to detect the protein expression levels of GPX4 and FTH1 in HCT116 and LS174T cells after DMF treatment. **H** Tumor growth analysis of HCT116 xenograft models in the NC vector group, DMF (10 mg/kg) group, and DMF (30 mg/kg) treatment group. Representative images of tumors and curves showing tumor volume changes over time are presented. **I** IHC was performed to examine Ki-67 staining in tumor tissues (scale bar: 50 μm). *n* = 3 biological replicates per group. Data are presented as mean ± SD. *p < 0.05, ***p < 0.001, ****p < 0.0001.

FH catalyzes the conversion of fumarate to malate, and its downregulation leads to the accumulation of its upstream metabolites fumarate and succinate. Metabolomic profiling of the Glu(−) and Glu(−) + FH(OE) groups confirmed that glucose starvation increased glycolytic end products (pyruvate, lactate) and several gluconeogenic metabolites, induced severe TCA cycle imbalance with abnormal accumulation of fumarate, succinate, succinyl-CoA, and acetyl-CoA, and decreased downstream intermediates including malate, oxaloacetate, and citrate. Notably, FH overexpression partially reversed these metabolic disturbances, indicating that FH downregulation is a key driver of glucose starvation-induced metabolic reprogramming (Fig. 4A). Cellular assays confirmed time-dependent increases in intracellular fumarate and succinate levels under prolonged glucose starvation (0, 12, 24, 48, 72 h) (Fig. 4B). In 68 clinical CRC patients, serum fumarate and succinate levels correlated with advanced clinical stage, with pronounced accumulation in T3-4 and lymph node metastasis (N+) patients (Fig. 4C).

Given that fumarate modulates ferroptosis by regulating intracellular free iron and redox balance, FH was restored using Lys05 under glucose starvation, followed by treatment with dimethyl fumarate (DMF) to mimic fumarate accumulation upon FH inactivation. DMF significantly reduced intracellular Fe²⁺ and ROS levels, upregulated GPX4 and FTH1 expression, and restored cell proliferation, partially reversing ferroptosis and proliferation inhibition caused by FH restoration (Fig. 4D–G). In vivo, DMF promoted tumor growth in a dose-dependent manner, with the high-dose DMF group exhibiting larger tumor volumes and higher Ki-67 expression (Fig. 4H, I).

Consistent with DMF, exogenous diethyl succinate (DES) similarly enhanced cell proliferation, upregulated GPX4, decreased FH protein levels without affecting FH enzymatic activity, and reduced intracellular Fe²⁺ and ROS levels, indicating that succinate accumulation also inhibits ferroptosis. In xenograft models, DES exerted dose-dependent tumor-promoting effects, accompanied by elevated Ki-67 expression and reduced FH protein levels in the high-dose DES group (Fig. S2A–G).

### Fumarate inhibits ferroptosis by enhancing NRF2 stability and upregulating GPX4 and FTH1 expression

**Fig. 5:**
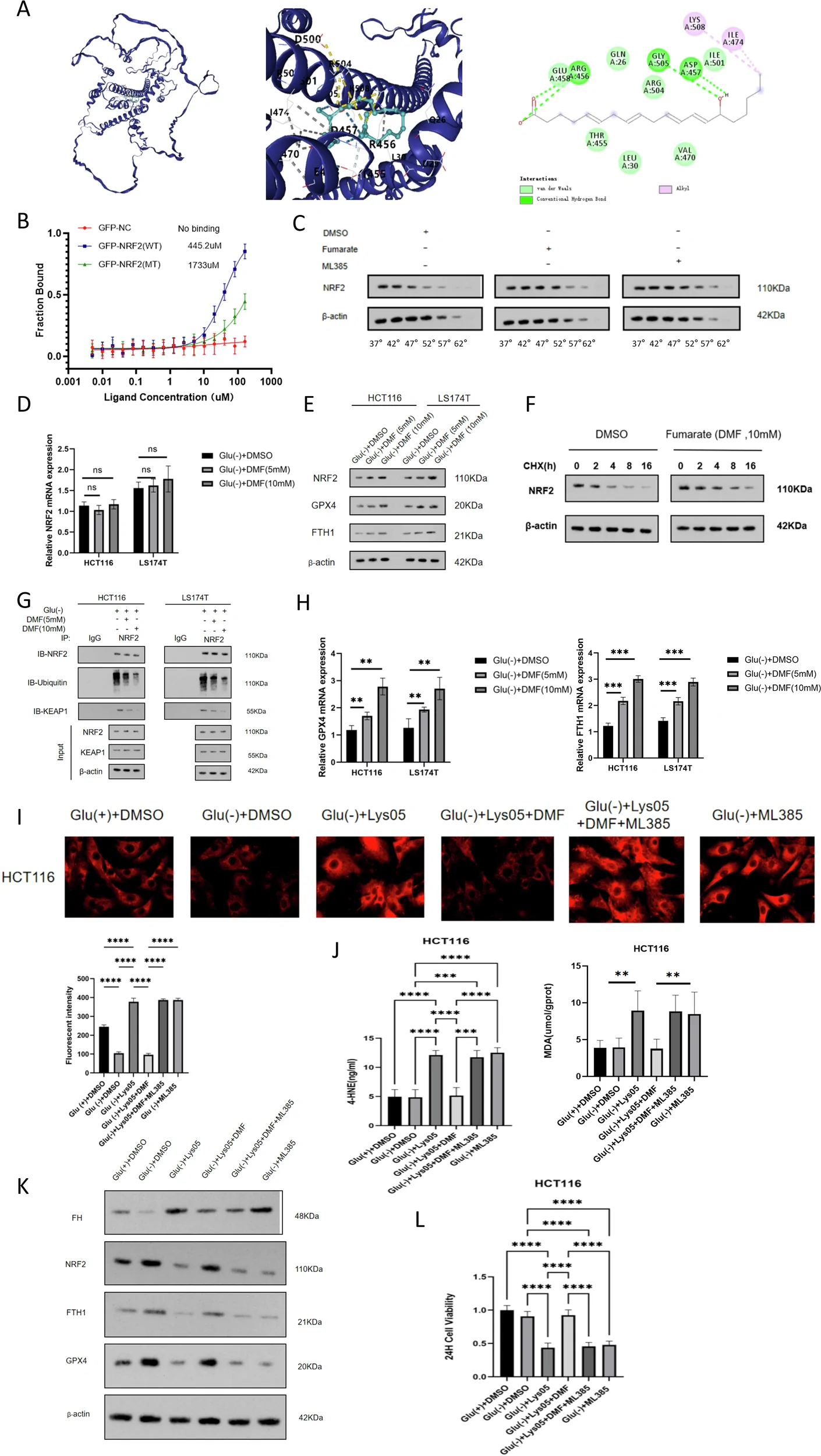
Fumarate inhibits ferroptosis by enhancing NRF2 stability and upregulating GPX4 and FTH1 expression. **A** Molecular docking analysis was performed to evaluate the interaction between fumarate and NRF2 protein. **B** Microscale thermophoresis (MST) assay was conducted to detect the binding capacity of fumarate (DMF) to GFP empty vector, wild-type GFP-NRF2 and GFP-NRF2 mutant. **C** Cellular thermal shift assay (CETSA) was used to detect the binding and thermostability between fumarate and NRF2. **D** qRT-PCR was performed to detect NRF2 mRNA expression in HCT116 and LS174T cells treated with different concentrations of DMF (0 mM, 5 mM, 10 mM). **E** Western blot was conducted to detect NRF2, GPX4, and FTH1 protein expression. **F** Western blot was conducted to examine NRF2 protein expression in HCT116 cells treated with CHX for different time periods in the presence or absence of DMF (10 mM). **G** Co-immunoprecipitation (Co-IP) assay was used to detect the interaction between KEAP1 and NRF2, as well as the changes in NRF2 ubiquitination modification, after treatment with different concentrations of DMF. **H** qRT-PCR was performed to detect GPX4 and FTH1 mRNA expression in CRC cells treated with different concentrations of DMF. **I** FerroOrange fluorescent probe was employed to detect intracellular Fe²⁺ levels in HCT116 cells treated with NRF2 inhibitor ML385 (scale bar: 20 μm). **J** ELISA was performed to detect the levels of lipid peroxidation markers (4-HNE, MDA) in HCT116 cells treated with ML385. **K** Western blot was conducted to detect GPX4 and FTH1 protein expression treated with ML385. **L** CCK-8 assay was performed to detect the proliferative capacity of HCT116 cells treated with ML385. *n* = 3 biological replicates per group. Data are presented as mean ± SD. ns, not significant, *p < 0.05, **p < 0.01, ***p < 0.001, ****p < 0.0001.

We observed that fumarate accumulation upregulates the negative ferroptosis regulators GPX4 and FTH1. Previous studies have shown that both GPX4 and FTH1 are regulated by the upstream transcription factor NRF2 [28, 29], and dimethyl fumarate (DMF) is an activator of NRF2 [30]. Molecular docking predicted stable binding of fumarate to NRF2 with multiple interacting residues (Fig. 5A). Microscale thermophoresis (MST) confirmed concentration-dependent binding of fumarate to wild-type GFP-NRF2, which was markedly attenuated in a GFP-NRF2 mutant; no specific binding was detected with GFP alone, supporting a specific interaction between fumarate and NRF2 (Fig. 5B). Cellular thermal shift assays (CETSA) demonstrated that fumarate treatment significantly improved NRF2 thermostability at high temperatures (Fig. 5C), indicating that fumarate specifically binds and stabilizes NRF2. Under physiological conditions, KEAP1 binds NRF2 and promotes its ubiquitin-dependent degradation, 388 maintaining low NRF2 levels [31]. Under glucose starvation, DMF treatment did not alter NRF2 mRNA but increased NRF2 protein in a dose-dependent manner (Fig. 5D, E). CHX chase assays confirmed that DMF elevates NRF2 by enhancing its protein stability (Fig. 5F). Mechanistically, increasing DMF concentrations gradually reduced the NRF2–KEAP1 interaction and NRF2 ubiquitination (Fig. 5G) and dose-dependently upregulated mRNA and protein levels of the NRF2 target genes GPX4 and FTH1 (Fig. 5E, H). Under Glu(−) + Lys05 + DMF conditions, the NRF2 inhibitor ML385 reactivated ferroptosis and suppressed proliferation (Fig. 5L), as shown by elevated Fe²⁺ (Fig. 5I), 4-HNE and MDA levels (Fig. 5J), and decreased GPX4 and FTH1 expression (Fig. 5K).

Collectively, these results indicate that under glucose starvation, fumarate disrupts the NRF2–KEAP1 interaction, prevents KEAP1-mediated ubiquitination-dependent degradation, stabilizes NRF2, and upregulates GPX4 and FTH1, ultimately inhibiting ferroptosis and sustaining CRC cell proliferation.

### CPT1A-mediated FH succinylation under glucose starvation maintains CRC cell proliferation by inhibiting ferroptosis

**Fig. 6:**
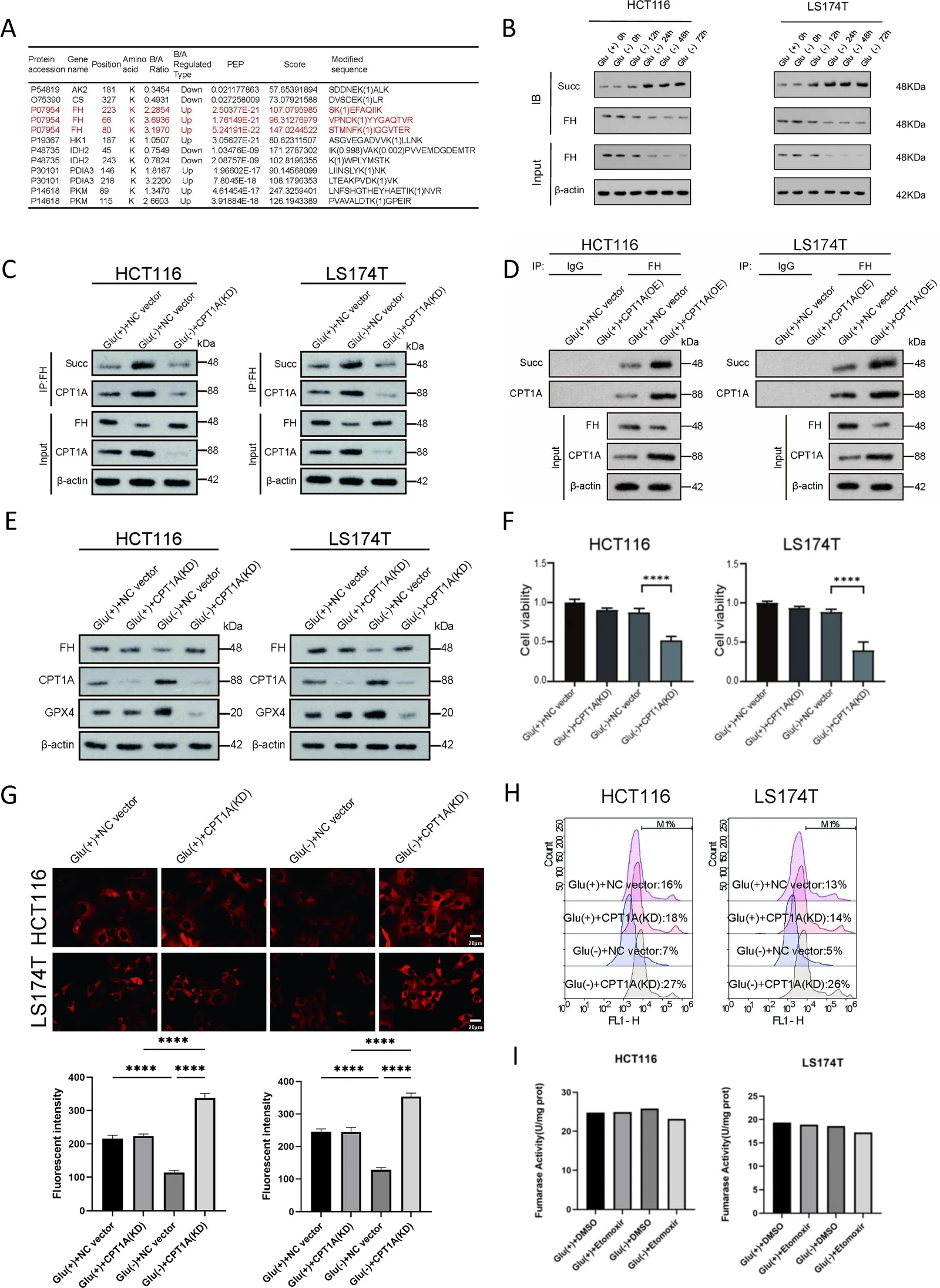
CPT1A-mediated FH succinylation under glucose starvation maintains CRC cell proliferation by inhibiting ferroptosis. **A** Succinyl-proteomic quantitative analysis was performed to detect the succinylation modification of FH protein in HCT116 cells. **B** Co-IP assay was conducted to detect FH protein and its succinylation modification levels at different time points (0, 12, 24, 48, 72 h) under glucose starvation conditions in HCT116 and LS174T cells. **C** After CPT1A knockdown, Co-IP assay was used to detect FH protein expression and its succinylation modification levels. **D** After CPT1A overexpression, Co-IP assay was performed to detect FH succinylation modification and protein expression levels. **E** After CPT1A knockdown, western blot was conducted to detect GPX4 protein expression levels. **F** After CPT1A knockdown, CCK-8 assay was used to evaluate cell proliferative capacity. **G** After CPT1A knockdown, FerroOrange fluorescent probe was used to perform immunofluorescent staining and quantitative analysis of intracellular Fe²⁺ levels (scale bar: 20 μm). **H** Flow cytometry was employed to detect lipid ROS levels using C11 BODIPY 581/591 staining. **I** After treating cells with the CPT1A enzyme inhibitor etomoxir sodium salt (ETO), FH-specific activity was measured by an FH enzyme activity assay. *n* = 3 biological replicates per group. Data are presented as mean ± SD. *p < 0.05, ***p < 0.001, ****p < 0.0001.

Our preliminary succinyl-proteomic profiling revealed elevated FH succinylation under glucose starvation (Fig. 6A). Co-IP confirmed time-dependent enhancement of FH succinylation, which was consistent with reduced FH protein (Fig. 6B). To identify the regulatory enzyme, known succinyltransferases (CPT1A, KAT2A, HAT1, EP300) were knocked down. Only CPT1A knockdown significantly reduced the FH-CPT1A interaction, decreased FH succinylation, and elevated FH protein expression (Fig. 6C). Knockdown of KAT2A, HAT1, or EP300 had no significant effects (Fig. S3A). Treatment with the CPT1A inhibitor etomoxir sodium salt (ETO) yielded similar results (Fig. S3B), indicating that glucose starvation induces CPT1A-mediated FH succinylation and downregulation. Under Glu(+) conditions, CPT1A overexpression enhanced its interaction with FH, increased FH succinylation, and reduced FH protein (Fig. 6D), further supporting that CPT1A is a key regulator of FH succinylation.

We next assessed the functional consequences of CPT1A-mediated FH succinylation on ferroptosis and cell proliferation. Under Glu(-) conditions, ETO treatment or CPT1A knockdown increased FH protein, decreased GPX4 expression (Figs. 6E and S3C), elevated intracellular Fe²⁺ and ROS levels (Figs. 6G, H and S3E, F), and suppressed cell proliferative activity (Figs. 6F and S3D); these effects were minor under Glu(+) conditions. Thus, CPT1A-mediated FH succinylation under glucose starvation maintains CRC cell proliferation by inhibiting ferroptosis. FH activity assays under varying glucose and ETO conditions confirmed stable specific activity regardless of glucose availability or CPT1A inhibition, indicating that CPT1A-mediated succinylation does not directly affect FH catalytic function (Fig. 6I).

### Under glucose starvation, FH succinylation at K66/K80 promotes autophagy-lysosome pathway degradation, inhibits ferroptosis, and sustains CRC cell proliferation

**Fig. 7:**
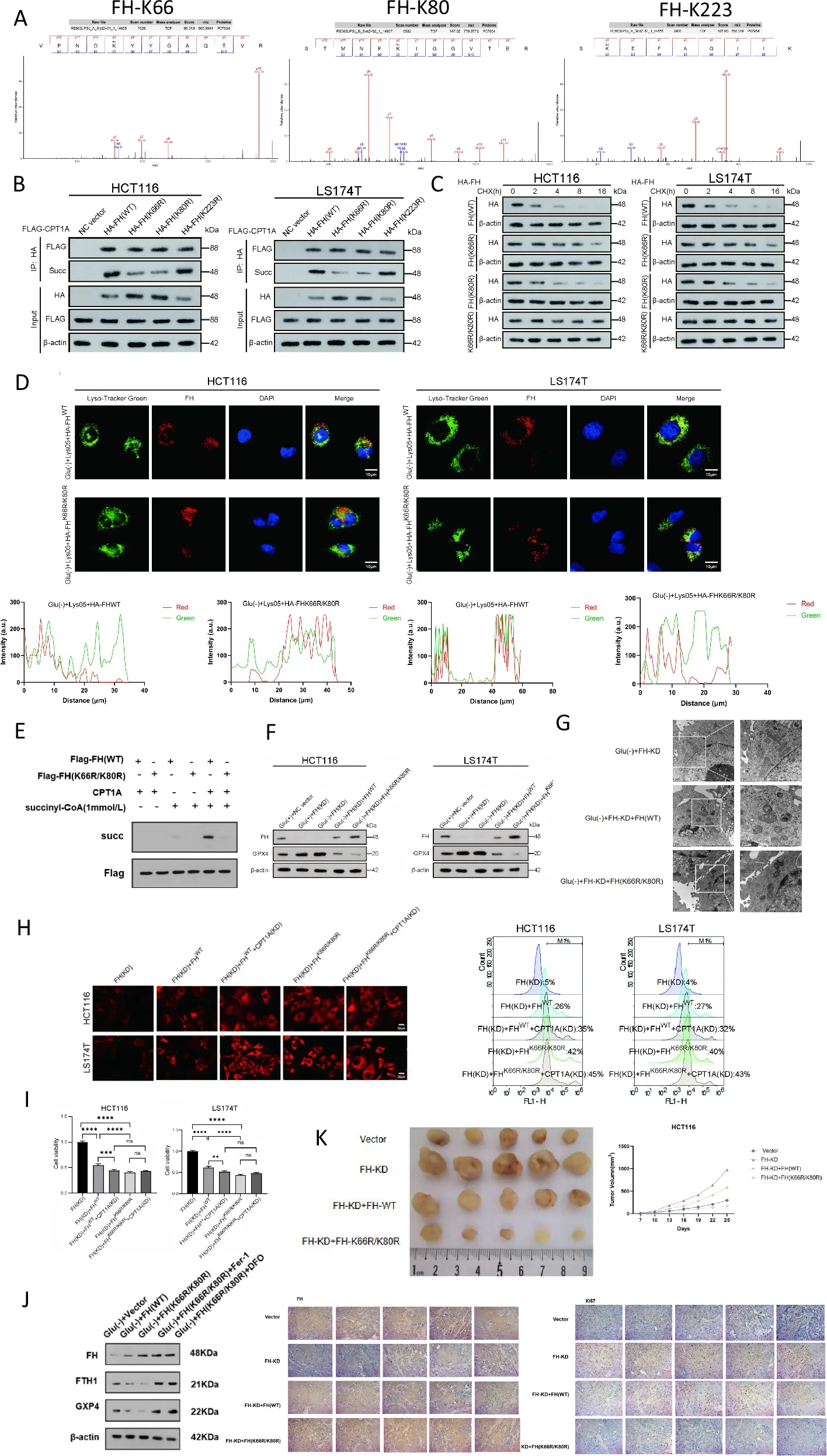
Under glucose starvation, FH succinylation at K66/K80 dual sites promotes FH degradation via the autophagy-lysosome pathway, inhibits ferroptosis, and maintains CRC cell proliferation. **A** Mass spectrometry was performed to detect potential succinylation modification sites of the FH protein under glucose starvation. **B** After transfection with exogenous FLAG-CPT1A, HA-FH (WT), HA-FH (K66R), HA-FH (K80R), and HA-FH (K223R) plasmids, 845 Co-IP assay was conducted to detect FH succinylation modification levels. **C** After transfection with HA-FH(WT), HA-FH(K66R), HA-FH(K80R), and HA-FH(K66R/K80R) plasmids, respectively, western blot was conducted to examine FH protein expression in CRC cells treated with CHX for different time periods (0, 2, 4, 8, 16 h) to evaluate FH protein stability. **D** Confocal microscopy was used to detect the colocalization of FH (red) and lysosomes (green) in Glu(-) + Lys05 + FH(WT) and Glu(-) + Lys05 + FH(K66R/K80R) groups (scale bar: 10 μm). Quantitative analysis of the FH protein and autophagy markers was also performed. **E** In vitro succinylation assay was used to detect the enzymatic succinylation activity of CPT1A on FH. **F** Western blot was conducted to detect GPX4 expression in Glu(+) + FH(KD) group, Glu(-) + FH(KD) group, Glu(-) + FH(KD) + FH(WT)group, and Glu(-) + FH(KD) + FH(K66R/K80R) group. **G** Transmission electron microscopy (TEM) showed the mitochondrial ultrastructure of HCT116 cells (scale bar: 2 μm). **H** CPT1A was further knocked down in the FH(KD) + FH(K66R/K80R) group. FerroOrange fluorescent probe was used to detect Fe²⁺ (scale bar: 20 μm), and C11 BODIPY 581/591 fluorescent probe was used for flow cytometry to detect ROS levels. **I** CCK-8 assay was performed to detect the proliferative capacity. **J** Fer-1 or DFO was added to cells treated with Glu(-) + FH(K66R/K80R). Western blot was conducted to detect FH, GPX4, and FTH1 protein expression. **K** Tumor growth analysis of xenograft models in the FH (KD) group, FH(KD) + FH(WT) group, and FH(KD) + FH(WT) + FH(K66R/K80R) group. Representative images of tumors and curves showing tumor volume changes over time are presented. Western blot was used to detect the expression of FH and GPX4 in xenograft tumor tissues, and IHC was performed to examine FH and Ki-67 expression (scale bar: 50 μm). *n* = 3 biological replicates per group. Data are presented as mean ± SD. ns, not significant, *p < 0.05, **p < 0.01, ***p < 0.001, ****p < 0.0001.

Mass spectrometry identified three potential succinylation sites in FH under glucose starvation: K66, K80, and K223 (Fig. 7A). To identify the key modification sites, our study mutated the potential succinylation site lysine (K) to arginine (R) to mimic desuccinylation, followed by Co-IP detection using HA antibody. Compared with wild-type FH, FH(K66R) and FH(K80R) single mutations significantly reduced FH succinylation and increased FH protein, whereas FH(K223R) had no effect, identifying K66 and K80 as the primary regulatory sites (Fig. 7B). CHX and MG132 treatment confirmed that FH(K66R) and FH(K80R) enhanced FH protein stability and suppressed its degradation, with synergistic effects observed in the FH(K66R/K80R) double mutant (Figs. 7C and S4A). In vitro succinylation assays showed robust succinylation signals in wild-type FH incubated with succinyl-CoA and CPT1A, which were markedly attenuated in the FH(K66R/K80R) double mutant, confirming that CPT1A specifically promotes FH succinylation at K66/K80 (Fig. 7E). Confocal microscopy showed enhanced colocalization of wild-type FH with lysosomes under glucose starvation, which was substantially reduced in the FH(K66R/K80R) mutant, indicating that K66/K80 succinylation facilitates FH degradation via the autophagy-lysosome pathway (Fig. 7D).

To verify that regulation of ferroptosis and proliferation by FH succinylation depends on K66/K80, endogenous FH was knocked down under Glu(-) conditions and replaced with the FH(K66R/K80R) mutant, resulting in decreased GPX4 (Fig. 7F), elevated Fe²⁺ and ROS (Fig. S4C), typical ferroptotic mitochondrial damage visualized by TEM (Fig. 7G), and accompanied by reduced CRC cell proliferation (Fig. S4B). Further knockdown of CPT1A in FH(KD) + FH(K66R/K80R) cells still resulted in enhanced ferroptosis and reduced cell proliferation (Figs. 7H, I and S4D). Rescue experiments using Fer-1 or DFO under Glu(−) + FH(K66R/K80R) conditions reduced 4-HNE and MDA (Fig. S4E), upregulated GPX4 and FTH1 (Fig. 7J), inhibited ferroptosis, and restored cell proliferation (Fig. S4E). In vivo xenograft studies showed that FH(KD) promoted tumor growth, FH(KD) + FH(WT) partially inhibited growth, and FH(KD) + FH(WT) + FH(K66R/K80R) exerted stronger antitumor effects with smaller tumor volumes, increased FH, and decreased Ki-67 (Fig. 7K). In summary, CPT1A-mediated FH succinylation at K66/K80 under glucose starvation inhibits ferroptosis and sustains CRC cell proliferation, whereas blocking succinylation at these sites markedly enhances ferroptosis and suppresses tumor growth.

## Discussion

Rapidly proliferating CRC cells frequently encounter glucose-deprived microenvironments, imposing metabolic stress that drives adaptive metabolic reprogramming to sustain proliferation and survival. FH, a key TCA cycle enzyme, is downregulated in multiple cancers and implicated in tumor proliferation and invasion [32, 33]. Consistent with these reports, we confirmed FH downregulation in CRC through public transcriptomic datasets and 68 paired clinical specimens, establishing a positive correlation between FH expression and overall survival and identifying FH as an independent protective factor in CRC. Functional assays further demonstrated that FH overexpression significantly suppresses cell proliferation and invasion, while FH knockdown exerts opposite effects, establishing FH as a tumor suppressor in CRC. In an in vitro glucose starvation model, we observed glucose concentration-dependent downregulation of FH protein without corresponding changes in mRNA, indicating post-translational regulatory mechanisms. Further investigation showed that CRC cell proliferation is inhibited in the early stage of glucose starvation, but cells gradually adapt and restore growth after 24 h.

Mechanistically, glucose starvation promoted FH degradation via the autophagy-lysosome pathway independently of the proteasome or mitophagy. Enzymatic activity assays confirmed that glucose starvation affects only FH protein abundance, not catalytic activity. Ferroptosis is an iron-dependent, lipid peroxidation-mediated form of cell death that plays a critical role in tumor proliferation and nutrient stress responses [34]. Previous studies reported that glucose starvation induces ferroptosis resistance in cancer cells, involving key transcription factors (NRF2, ATF4) and effector proteins (SLC7A11, GPX4) [35–39]. Our study demonstrates that ferroptosis was inhibited in the early stage of glucose starvation, with maximal suppression observed at 24 h persisting through 72 h, as evidenced by upregulated GPX4 expression and decreased levels of intracellular Fe²⁺, lipid peroxidation products (4-HNE, MDA), and lipid ROS. This ferroptosis suppression supported CRC cell survival and coincided temporally with the gradual adaptation of cells to glucose deprivation and recovery of proliferative capacity after 24 h. Importantly, restoring FH expression under glucose starvation reversed ferroptosis resistance, with typical ferroptotic mitochondrial alterations observed by TEM. The ferroptosis inhibitors Fer-1 and DFO rescued proliferation inhibition induced by FH overexpression, and in vivo experiments confirmed that Fer-1 partially reverses FH-mediated tumor growth inhibition. Collectively, these findings establish glucose starvation-induced FH downregulation as a critical mechanism enabling CRC cells to evade ferroptosis and sustain proliferation, positioning FH as a potential metabolic intervention target in CRC [40].

Our metabolomic profiling revealed that glucose starvation disrupts the TCA cycle, most notably the accumulation of the upstream metabolites fumarate and succinate in a time-dependent manner. Restoring FH partially reverses these metabolic disturbances, indicating that FH downregulation is a key driver of TCA cycle imbalance under glucose starvation. Exogenous supplementation with the fumarate analog DMF or the succinate analog DES effectively inhibited ferroptosis and promoted CRC cell proliferation. Clinical correlation analyses further demonstrated that elevated fumarate and succinate levels were associated with advanced tumor stage and poor prognosis, suggesting their utility as prognostic biomarkers in CRC. Mechanistically, we demonstrate that accumulated fumarate directly binds NRF2, as evidenced by molecular docking, MST, and CETSA, enhancing its thermal stability. Fumarate disrupts the NRF2–KEAP1 interaction, thereby inhibiting KEAP1-mediated NRF2 ubiquitination and degradation, leading to NRF2 stabilization and transcriptional upregulation of downstream targets GPX4 and FTH1, ultimately inhibiting ferroptosis and sustaining CRC cell proliferation. Our findings provide new mechanistic insights into ferroptosis regulation under glucose starvation and expand the molecular paradigm by which metabolites participate in tumor stress adaptation [30, 41, 42]. Previous studies reported that FH mutation or inactivation leads to abnormal accumulation of small metabolites such as fumarate and succinate, which activate oncogenic signaling pathways and are termed oncometabolites [43, 44]. Targeting or blocking oncometabolites represents a promising novel direction in cancer therapy. Notably, DMF is an FDA-approved therapeutic for multiple sclerosis and has demonstrated antitumor activity in diffuse large B-cell lymphoma and other hematologic malignancies [45–48]. Our results indicate that DMF promotes CRC cell survival by inhibiting ferroptosis. As a clinically approved drug with established safety, DMF may be repurposed in combination with ferroptosis inducers or FH modulators to reverse its pro-proliferative effects in CRC, fully exploiting its metabolic intervention potential for precision metabolic therapy. Emerging evidence implicates aberrant protein succinylation in cancer cell adaptation to nutrient deprivation stress, modulating key metabolic enzyme stability and activity to drive metabolic reprogramming and support rapid proliferation [49–52]. For example, succinylation of PKM2 and ME2 contributes to adaptive tumor growth under nutrient deficiency in CRC cells [26, 53]. Our preliminary succinyl-proteomic profiling showed globally increased protein succinylation under glucose starvation, most notably on FH, in a time-dependent manner. Further investigation revealed that CPT1A is the primary succinyltransferase mediating FH succinylation under glucose starvation, specifically at residues K66 and K80. Functional experiments confirmed that the FH(K66R/K80R) double mutation markedly enhances FH protein stability, blocks the autophagy-lysosome pathway degradation, increases FH expression, and consequently promotes ferroptosis while suppressing CRC cell proliferation. In vitro succinylation assays further verified that CPT1A specifically promotes FH succinylation at K66/K80 without affecting its catalytic activity. A recent study reported O-GlcNAcylation of FH at Ser75 under glucose starvation, which promotes tumor cell proliferation by disrupting FH-ATF2 interaction [54], suggesting that FH integrates diverse PTM networks to orchestrate multilayered stress adaptation, underscoring its functional versatility in tumor biology.

In conclusion, this study delineates a novel regulatory mechanism by which CRC cells adapt to glucose-deprived microenvironments: glucose starvation induces CPT1A-mediated succinylation of FH at K66/K80, promoting FH degradation via the autophagy-lysosome pathway and reducing FH expression. FH downregulation disrupts TCA cycle homeostasis and causes abnormal accumulation of fumarate and succinate. Accumulated fumarate directly binds NRF2, disrupts the NRF2–KEAP1 interaction, inhibits NRF2 ubiquitination, enhances its stability, and activates downstream GPX4 and FTH1 to inhibit ferroptosis, ultimately sustaining CRC cell proliferation under metabolic stress. Our findings provide new insights into ferroptosis regulation under tumor metabolic stress and establish a core regulatory axis: Glucose starvation-CPT1A-mediated FH succinylation-fumarate-NRF2-ferroptosis. Targeting this axis may offer novel strategies for CRC treatment, providing both a theoretical framework and experimental foundation for future therapeutic development.

## Supplementary information

## Data Availability

The datasets generated and/or analyzed during the current study are available from the corresponding author upon reasonable request.
