# Supplementary material for: Glucose starvation induces fumarate hydratase succinylation and inhibits ferroptosis to maintain colon cancer cell proliferation
Source: Cell Death Dis. 2026 Jun 22;17(1):773. doi: 10.1038/s41419-026-08975-9 (PMC13538377; doi:10.1038/s41419-026-08975-9)
Supplement: Supplementary file 1 [file 41419_2026_8975_MOESM1_ESM.docx]

**Supplementary Table 1.**

| Name | Sequence |
| --- | --- |
| FH(forward) | 5’-GGAGGTGTGACAGAACGCAT-3’ |
| FH(reverse) | 5’-CATCTGCTGCCTTCATTATTGC-3’ |
| GPX4(forward) | 5’-GAGGCAAGACCGAAGTAAACTAC-3’ |
| GPX4(reverse) | 5’-CCGAACTGGTTACACGGGAA-3’ |
| NRF2(forward) | 5’-TTCCCGGTCACATCGAGAG-3’ |
| NRF2(reverse) | 5’-TCCTGTTGCATACCGTCTAAATC-3’ |
| FTH1(forward) | 5’-TGAAGCTGCAGAACCAACGAGG-3’ |
| FTH1(reverse) | 5’-GCACACTCCATTGCATTCAGCC-3’ |
| β-actin(forward) | 5’-CATTAAGGAGAAGCTGTGCT-3’ |
| β-actin(reverse) | 5’-GTTGAAGGTAGTTTCGTGGA |

**Supplementary Table 2.**

| Primary antibodies | Dilution | Company Catalog | Secondary antibodies | Dilution |
| --- | --- | --- | --- | --- |
| FH | 1:1000 | Ptgcn, 11375-1-AP | Goat Anti Rabbit IgG/HRP | 1:4000 |
| GPX4 | 1:500 | Sab, #32506 | Goat Anti Rabbit IgG/HRP | 1:4000 |
| FTH1 | 1:500 | Abcam, ab75972 | Goat Anti Rabbit IgG/HRP | 1:4000 |
| SLC7A11 | 1:1000 | Abcam, ab175186 | Goat Anti Rabbit IgG/HRP | 1:4000 |
| CPT1A | 1:1000 | Abcam, ab234906 | Goat Anti Rabbit IgG/HRP | 1:4000 |
| KAT2A | 1:200 | Omnimabs, OM155843 | Goat Anti Rabbit IgG/HRP | 1:4000 |
| EP300 | 1:1000 | Abcam, ab275378 | Goat Anti Rabbit IgG/HRP | 1:4000 |
| HAT1 | 1:1000 | Ptgcn, 11432-1-AP | Goat Anti Rabbit IgG/HRP | 1:4000 |
| KEAP1 | 1:500 | Abcam, ab139729 | Goat Anti Rabbit IgG/HRP | 1:4000 |
| NRF2 | 1:1000 | abclonal ,A1244 | Goat Anti Rabbit IgG/HRP | 1:4000 |
| GSDMD | 1:1000 | Ptgcn, 20770-1-AP; | Goat Anti Rabbit IgG/HRP | 1:4000 |
| Beclin-1 | 1:1000 | Ptgcn ,11306-1-AP | Goat Anti Rabbit IgG/HRP | 1:4000 |
| Bcl-2 | 1:2000 | Ptgcn,80313-1-RR, | Goat Anti Rabbit IgG/HRP | 1:4000 |
| P62/SQSTM1 | 1:1600 | Ptgcn,84826-1-RR | Goat Anti Rabbit IgG/HRP | 1:4000 |
| Caspase-3 | 1:500 | Ptgcn, 82202-1-RR | Goat Anti Rabbit IgG/HRP | 1:4000 |
| Caspase-1 | 1:800 | Ptgcn,22915-1-AP | Goat Anti Rabbit IgG/HRP | 1:4000 |
| Ubiquitin | 1:200 | Ptgcn,10201-2-AP | Goat Anti Rabbit IgG/HRP | 1:4000 |
| Succinyllysine | 1:500 | PTM,PTM-401 | Goat Anti Rabbit IgG/HRP | 1:4000 |
| HA | 1:2000 | Ptgcn,51064-2-AP | Goat Anti Rabbit IgG/HRP | 1:4000 |
| FLAG | 1:1000 | Abcam,ab205606 | Goat Anti Rabbit IgG/HRP | 1:4000 |
| β-actin | 1:2000 | Ptgcn, 66009-1-Ig | Goat Anti Mouse IgG/HRP | 1:4000 |

**Supplementary Table 3.**

| SiRNA/ShRNA | Sequence |
| --- | --- |
| siRNA-FH-1 | 5′-GAAUCCAGGCCAAUACAGAATT-3′ |
| siRNA-FH-2 | 5′-CGGAUAAGUUUGAAAGGAATT-3′ |
| siRNA-NC-1 | 5′-UUCUCCGAACGUGUCACGU-3′ |
| siRNA-CPT1A-1 | 5′-CCCCTCGTTAGGAATAATT-3′ |
| siRNA-CPT1A-2 | 5′-AGTCGTTCACGTTTGTTGT-3′ |
| siRNA-CPT1A-3 | 5′-GCCTTTACGTGGTGTCTAA-3′ |
| siRNA-NC-2 | 5′-TTCTCCGAACGTGTCACGT-3′ |
| shRNA-KAT2A-1 | 5’-CCGGGCGCATGCCTAAGGAGTATATCTCGAGATAT  ACTCCTTAGGCATGCGCTTTTTG-3′ |
| shRNA-KAT2A-2 | 5′-CCGGGGCTACCTACAAGGTCAATTACTCGAGTAATT  GACCTTGTAGGTAGCCTTTTTG-3′ |
| siRNA-HAT1 | 5′-GAAGCTACAGACTGGATATTA-3′ |
| siRNA-EP3001-1 | 5′-ATACTCAGCCGGAGGATATTT-3′ |
| siRNA-EP3001-2 | 5′-TAACCAATGGTGGTGATATTA-3′ |

**Supplementary Table 4.**

| Antibodies | Company |
| --- | --- |
| Anti-IgG | Proteintech |
| Anti-Succinyllysine | Jingjie Biotech |
| Anti-HA | Beyotime |
| Anti-Flag | Yaji Bio |

**Supplementary Table 5.**

**Experimental grouping of HCT116 and LS174T cells under glucose deprivation**

| Group | Treatment | Replicate 1 | Replicate 2 | Replicate 3 | Mean | Relative fluorescence intensity |
| --- | --- | --- | --- | --- | --- | --- |
| HCT116 | | | | | | |
| Group A | Glu+(0h) | 245.40 | 255.90 | 265.10 | 255.47 | 1.00 |
| Group B | Glu−(0h) | 239.50 | 243.10 | 257.80 | 246.80 | 0.97 |
| Group C | Glu−(12h) | 98.50 | 104.50 | 107.20 | 103.40 | 0.40 |
| Group D | Glu−(24h) | 100.40 | 112.20 | 103.50 | 105.37 | 0.41 |
| Group E | Glu−(48h) | 97.60 | 108.50 | 116.30 | 107.47 | 0.42 |
| Group F | Glu−(72h) | 96.20 | 95.40 | 106.40 | 99.33 | 0.40 |
| LS174T | | | | | | |
| Group A | Glu+(0h) | 231.20 | 228.90 | 243.10 | 234.40 | 1.00 |
| Group B | Glu−(0h) | 224.70 | 234.60 | 240.60 | 233.30 | 1.00 |
| Group C | Glu−(12h) | 86.30 | 99.40 | 90.20 | 91.97 | 0.39 |
| Group D | Glu−(24h) | 89.00 | 98.30 | 94.20 | 93.83 | 0.40 |
| Group E | Glu−(48h) | 86.40 | 82.30 | 97.10 | 88.60 | 0.38 |
| Group F | Glu−(72h) | 90.30 | 92.50 | 88.10 | 90.30 | 0.39 |

**Experimental grouping of HCT116 and LS174T cells treated with diethyl succinate (DES)**

| Group | Treatment | Replicate 1 | Replicate 2 | Replicate 3 | Mean | Relative fluorescence intensity |
| --- | --- | --- | --- | --- | --- | --- |
| HCT116 | | | | | | |
| Group A | DMSO | 256.40 | 238.70 | 241.40 | 245.50 | 1.00 |
| Group B | DES(10nM) | 103.60 | 121.60 | 113.20 | 112.80 | 0.46 |
| LS174T | | | | | | |
| Group A | DMSO | 255.30 | 239.40 | 236.70 | 243.80 | 1.00 |
| Group B | DES(10nM) | 98.50 | 99.70 | 87.60 | 95.27 | 0.39 |

**Experimental grouping for fumarate–NRF2 pathway analysis under glucose deprivation**

| Group | Treatment | Replicate 1 | Replicate 2 | Replicate 3 | Mean | Relative fluorescence intensity |
| --- | --- | --- | --- | --- | --- | --- |
| HCT116 | | | | | | |
| Group A | Glu (+)+DMSO | 236.10 | 245.40 | 254.70 | 245.40 | 1.00 |
| Group B | Glu (-)+DMSO | 112.90 | 99.40 | 102.30 | 104.87 | 0.43 |
| Group C | Glu (-)+Lys05 | 387.60 | 355.70 | 389.00 | 377.43 | 1.54 |
| Group D | Glu (-)+Lys05+DMF | 97.70 | 89.40 | 102.80 | 96.63 | 0.39 |
| Group E | Glu(-)+Lys05+DMF+ML385 | 378.90 | 388.00 | 392.10 | 386.33 | 1.57 |
| Group F | Glu (-)+ML385 | 387.80 | 395.30 | 376.60 | 386.57 | 1.58 |

**Experimental grouping for FH restoration under glucose deprivation**

| Group | Treatment | Replicate 1 | Replicate 2 | Replicate 3 | Mean | Relative fluorescence intensity |
| --- | --- | --- | --- | --- | --- | --- |
| HCT116 | | | | | | |
| Group A1 | Glu(+)+DMSO | 213.7 | 198.5 | 234.6 | 215.60 | 1.00 |
| Group A2 | Glu(+)+Lys05 | 196.4 | 208.6 | 222.4 | 209.13 | 0.97 |
| Group B1 | Glu(-)+DMSO | 112.8 | 99.7 | 108.8 | 107.10 | 0.50 |
| Group B2 | Glu(-)+Lys05 | 365.3 | 344.0 | 356.1 | 355.13 | 1.65 |
| LS174T | | | | | | |
| Group A1 | Glu(+)+DMSO | 246.0 | 259.8 | 237.4 | 247.73 | 1.00 |
| Group A2 | Glu(+)+Lys05 | 256.2 | 260.4 | 249.6 | 255.40 | 1.03 |
| Group B1 | Glu(-)+DMSO | 101.5 | 124.1 | 116.0 | 113.87 | 0.46 |
| Group B2 | Glu(-)+Lys05 | 389.2 | 376.5 | 370.5 | 378.73 | 1.53 |

**Experimental grouping for FH-dependent regulation of ferroptosis under glucose deprivation**

| Group | Treatment | Replicate 1 | Replicate 2 | Replicate 3 | Mean | Relative fluorescence intensity |
| --- | --- | --- | --- | --- | --- | --- |
| HCT116 | | | | | | |
| Group A | Glu(+)+DMSO | 218.9 | 222.7 | 190.5 | 210.70 | 1.00 |
| Group B | Glu(-)+DMSO | 114.9 | 111.6 | 105.5 | 110.67 | 0.53 |
| Group C | Glu(-)+Lys05 | 339.1 | 342.3 | 338.7 | 340.03 | 1.61 |
| Group D | Glu(-)+Lys05+DMF | 100.4 | 106.7 | 113.3 | 106.80 | 0.51 |
| LS174T | | | | | | |
| Group A | Glu(+)+DMSO | 230.7 | 251.6 | 240.2 | 240.83 | 1.00 |
| Group B | Glu(-)+DMSO | 114.8 | 123.1 | 108.9 | 115.60 | 0.48 |
| Group C | Glu(-)+Lys05 | 335.8 | 343.0 | 369.2 | 349.33 | 1.45 |
| Group D | Glu(-)+Lys05+DMF | 99.4 | 115.3 | 102.6 | 105.77 | 0.44 |

**Experimental grouping for CPT1A inhibition under glucose deprivation**

| Group | Treatment | Replicate 1 | Replicate 2 | Replicate 3 | Mean | Relative fluorescence intensity |
| --- | --- | --- | --- | --- | --- | --- |
| HCT116 | | | | | | |
| Group A1 | Glu(+)+DMSO | 207.3 | 224.1 | 231.6 | 221.00 | 1.00 |
| Group A2 | Glu(+)+Etomoxir | 225.5 | 220.8 | 208.4 | 218.23 | 0.99 |
| Group B1 | Glu(-)+DMSO | 120.3 | 103.7 | 112.2 | 112.07 | 0.51 |
| Group B2 | Glu(-)+Etomoxir | 298.3 | 322.3 | 308.7 | 309.77 | 1.40 |
| LS174T | | | | | | |
| Group A1 | Glu(+)+DMSO | 222.8 | 235.2 | 233.1 | 230.37 | 1.00 |
| Group A2 | Glu(+)+Etomoxir | 213.8 | 227.6 | 224.0 | 221.80 | 0.96 |
| Group B1 | Glu(-)+DMSO | 113.8 | 102.6 | 109.5 | 108.63 | 0.47 |
| Group B2 | Glu(-)+Etomoxir | 344.2 | 324.1 | 339.3 | 335.87 | 1.46 |

**Experimental grouping for CPT1A knockdown under glucose deprivation**

| Group | Treatment | Replicate 1 | Replicate 2 | Replicate 3 | Mean | Relative fluorescence intensity |
| --- | --- | --- | --- | --- | --- | --- |
| HCT116 | | | | | | |
| Group A1 | Glu(+)+DMSO | 211.5 | 226.8 | 209.1 | 215.80 | 1.00 |
| Group A2 | Glu(+)+CPT1A(KD) | 223.8 | 229.2 | 217.0 | 223.33 | 1.03 |
| Group B1 | Glu(-)+DMSO | 109.3 | 111.4 | 121.5 | 114.07 | 0.53 |
| Group B2 | Glu(-)+CPT1A(KD) | 323.9 | 335.7 | 352.2 | 337.27 | 1.56 |
| LS174T | | | | | | |
| Group A1 | Glu(+)+DMSO | 243.6 | 238.2 | 255.1 | 245.63 | 1.00 |
| Group A2 | Glu(+)+CPT1A(KD) | 229.4 | 253.7 | 251.2 | 244.77 | 1.00 |
| Group B1 | Glu(-)+DMSO | 120.4 | 134.2 | 129.3 | 127.97 | 0.52 |
| Group B2 | Glu(-)+CPT1A(KD) | 365.6 | 344.0 | 349.6 | 353.07 | 1.44 |

**Experimental grouping for FH K66/K80 mutation under glucose deprivation**

| Group | Treatment | Replicate 1 | Replicate 2 | Replicate 3 | Mean | Relative fluorescence intensity |
| --- | --- | --- | --- | --- | --- | --- |
| HCT116 | | | | | | |
| Group A | Glu(+)+vector | 204.4 | 214.2 | 223.0 | 213.87 | 1.00 |
| Group B | Glu(+)/FH(KD) | 133.6 | 125.7 | 120.9 | 126.73 | 0.59 |
| Group C | Glu(-)/FH(KD) | 119.3 | 126.5 | 132.1 | 125.97 | 0.59 |
| Group D | Glu(-)/FH(KD)  +FH(WT) | 224.8 | 220.1 | 205.2 | 216.70 | 1.01 |
| Group E | Glu(-)/FH(KD)  +FH(K66R/K80R) | 326.8 | 334.1 | 327.2 | 329.37 | 1.54 |
| LS174T | | | | | | |
| Group A | Glu(+)+vector | 241.4 | 254.3 | 235.7 | 243.80 | 1.00 |
| Group B | Glu(+)/FH(KD) | 117.7 | 129.6 | 133.3 | 126.87 | 0.52 |
| Group C | Glu(-)/FH(KD) | 132.8 | 124.6 | 118.7 | 125.37 | 0.51 |
| Group D | Glu(-)/FH(KD)  +FH(WT) | 256.3 | 241.9 | 231.1 | 243.10 | 1.00 |
| Group E | Glu(-)/FH(KD)  +FH(K66R/K80R) | 367.7 | 355.0 | 374.2 | 365.63 | 1.50 |

**Experimental validation of CPT1A-mediated FH succinylation in ferroptosis regulation**

| Group | Treatment | Replicate 1 | Replicate 2 | Replicate 3 | Mean | Relative fluorescence intensity |
| --- | --- | --- | --- | --- | --- | --- |
| HCT116 | | | | | | |
| Group A | Glu(-)/FH(KD) | 98.7 | 111.9 | 113.4 | 108.00 | 1.00 |
| Group B | Glu(-)/FH(KD)  +FH(WT) | 187.5 | 201.5 | 197.6 | 195.53 | 1.81 |
| Group C | Glu(-)/FH(KD)  +FH(WT)+CPT1A(KD | 321.6 | 303.0 | 289.5 | 304.70 | 2.82 |
| Group D | Glu(-)/FH(KD)  +FH(K66R/K80R) | 292.4 | 311.3 | 291.1 | 298.27 | 2.76 |
| Group E | Glu(-)/FH(KD)  +FH(K66R/K80R)  +CPT1A(KD) | 323.6 | 306.7 | 296.1 | 308.80 | 2.86 |
| LS174T | | | | | | |
| Group A | Glu(-)/FH(KD) | 113.5 | 118.0 | 99.3 | 110.27 | 1.00 |
| Group B | Glu(-)/FH(KD)  +FH(WT) | 188.7 | 193.5 | 211.6 | 197.93 | 1.80 |
| Group C | Glu(-)/FH(KD)  +FH(WT)+CPT1A(KD | 287.7 | 279.8 | 293.1 | 286.87 | 2.60 |
| Group D | Glu(-)/FH(KD)  +FH(K66R/K80R) | 299.0 | 286.3 | 274.4 | 286.57 | 2.60 |
| Group E | Glu(-)/FH(KD)  +FH(K66R/K80R)  +CPT1A(KD) | 288.5 | 286.1 | 294.3 | 289.63 | 2.63 |
