# Supplementary material for: Glucose starvation induces fumarate hydratase succinylation and inhibits ferroptosis to maintain colon cancer cell proliferation
Source: Cell Death Dis. 2026 Jun 22;17(1):773. doi: 10.1038/s41419-026-08975-9 (PMC13538377; doi:10.1038/s41419-026-08975-9)
Supplement: Supplementary file 2 [file 41419_2026_8975_MOESM2_ESM.docx]

**Fig. S1 The effect of glucose starvation and ferroptosis inhibitors on cell viability and lipid peroxidation in CRC cells.**

A. Western blot was performed to detect FH expression in various CRC cell lines Caco-2, RKO, HT29, HCT116, CQLD205, T84, LS174T, and LS180). B.Cell viability and levels of lipid peroxidation markers (4-HNE, MDA) were detected in LS174T and HCT116 cells under different treatment conditions.

**Top row:** Cell viability was measured by CCK-8 assay; **Middle row:** Levels of 4-HNE were detected by ELISA; **Bottom row:** Levels of MDA were detected by ELISA. n = 3 biological replicates per group; Data are presented as mean ± SD. *P < 0.05, ***P < 0.001, ****P < 0.0001).

**Fig. S2 Abnormal accumulation of succinate inhibits ferroptosis and maintains CRC cell proliferation.**

A. CCK-8 assay was performed to detect the proliferative capacity of HCT116 and LS174T cells after DES (10 nM) treatment. B. FerroOrange fluorescent probe was used to perform immunofluorescent staining and quantitative analysis of intracellular Fe²⁺ levels (scale bar: 20μm). C. Flow cytometry was employed to detect lipid ROS levels using C11 BODIPY 581/591 staining. D. Western blot was performed to detect the protein expression levels of FH and GPX4. E. FH specific activity was measured by an FH enzyme activity assay after DES treatment. F. Tumor growth analysis of HCT116 xenograft models in the NC vector group, DES (10 mg/kg) group, and DES (30mg/kg) group. Representative images of tumors and curves showing tumor volume changes over time are presented. G. IHC was performed to examine Ki-67 staining in tumor tissues (scale bar: 50 μm). n = 3 biological replicates per group; Data are presented as mean ± SD. *P < 0.05, ***P < 0.001, ****P < 0.0001.

**Fig. S3 Evaluation of the effect of CPT1A-mediated FH succinylation on ferroptosis** **and cell proliferation by ETO treatment.**

A. After knockdown of KAT2A, HAT1, and EP300, Co-IP assay was used to detect FH protein expression and its succinylation modification levels. B. After treatment with the	CPT1A	enzyme inhibitor (ETO), Co-IP assay was conducted to detect FH succinylation modification and protein expression levels. C. After ETO treatment,

Western blot was conducted to detect GPX4 protein expression levels. D. After ETO treatment, CCK-8 assay was used to evaluate cell proliferative capacity. E. After ETO treatment, FerroOrange fluorescent probe was used to perform immunofluorescent staining and quantitative analysis of intracellular Fe²⁺ levels (scale bar: 20μm). F. After ETO treatment, Flow cytometry was employed to detect lipid ROS levels using C11 BODIPY 581/591 staining. n = 3 biological replicates per group; Data are presented as mean ± SD. *P < 0.05, ***P < 0.001, ****P < 0.0001.

**Fig. S4 Under glucose starvation, CPT1A-mediated succinylation modification of FH** **at K66/K80 sites inhibits ferroptosis and maintains CRC cell proliferation.**

After transfection with HA-FH(WT), HA-FH(K66R), HA-FH(K80R), and HA-FH(K66R/K80R) plasmids respectively, Western blot was conducted to examine FH protein expression treated with MG132 for different time periods (0, 2, 4, 8, 16h) to evaluate the degradation pathway of FH protein. B. CCK-8 assay was performed to detect the proliferative capacity	in Glu(+)+FH(KD) group, Glu(-)+FH(KD) group, Glu(-)+FH(KD)+FH(WT) group, and	Glu(-)+FH(KD)+FH(K66R/K80R) group. C. FerroOrange fluorescent probe was used to detect Fe²⁺ (scale bar: 20 μm), and C11 BODIPY 581/591 fluorescent probe was used for flow cytometry to detect ROS levels. D. CPT1A was further knocked down in the FH(KD)+FH(K66R/K80R) group, Co-IP assay was conducted to detect succinylation modification of FH at K66/K80 sites and the expression levels of FH and GPX4 proteins. E. Fer-1 or DFO was added to cells treated with Glu(-)+FH(K66R/K80R), CCK-8 and ELISA assay were performed to detect the proliferative capacity and the levels of lipid peroxidation markers (4-HNE, MDA).
