# Supplementary material for: Glucose starvation induces fumarate hydratase succinylation and inhibits ferroptosis to maintain colon cancer cell proliferation
Source: Cell Death Dis. 2026 Jun 22;17(1):773. doi: 10.1038/s41419-026-08975-9 (PMC13538377; doi:10.1038/s41419-026-08975-9)
Supplement: Supplementary file 3 [file 41419_2026_8975_MOESM7_ESM.docx]

**1.Clinical Detection of FH Expression（Figure 1 E）**

A total of 10 paired samples of colorectal cancer tissues and adjacent normal colorectal tissues were collected.

Western blotting (WB), quantitative PCR (qPCR), and immunohistochemistry (IHC) were performed to detect and compare FH expression levels between tumor and normal tissues.

T	N

FH

β-actin

|  | 1 | 2 | 3 | 4 | 5 | 6 | 7 | 8 | 9 | 10 |
| --- | --- | --- | --- | --- | --- | --- | --- | --- | --- | --- |
| FH-T | 25.06 | 21.46 | 124.95 | 38.71 | 16.85 | 185.70 | 60.24 | 18.62 | 0.00 | 38.78 |
| FH-N | 80.63 | 30.75 | 221.64 | 75.05 | 20.43 | 229.25 | 64.61 | 24.20 | 99.52 | 228.08 |
| β-actin-T | 151.32 | 256.98 | 312.33 | 330.52 | 311.58 | 381.93 | 223.05 | 315.31 | 341.52 | 188.88 |
| β-actin-N | 341.32 | 326.72 | 273.79 | 323.25 | 182.75 | 188.07 | 73.87 | 221.16 | 172.52 | 190.54 |

**2.Effect of Glucose Starvation on FH Expression （Figure 2 B）**

Colorectal cancer cell lines HCT-116 and LS174T were subjected to glucose starvation treatment with graded glucose concentrations (0, 1, and 5 mM), where 0 mM represented the glucose starvation group.

Western blotting (WB) and qPCR were performed to examine FH protein and mRNA expression levels, respectively.

Results showed that FH protein levels decreased progressively with the reduction of glucose concentration, whereas FH mRNA levels remained largely unchanged.

Grouping of HCT-116 and LS174T cells:

Group A: Cells treated with 0 mM glucose (Glucose starvation group)

Group B: Cells treated with 1 mM glucose

Group C: Cells treated with 5 mM glucose

FH	β-actin

|  | HCT-116 | | | LS174T | | |
| --- | --- | --- | --- | --- | --- | --- |
|  | A | B | C | A | B | C |
| FH | 13.09 | 29.04 | 73.57 | 23.86 | 40.91 | 54.32 |
|  | 22.12 | 38.24 | 73.41 | 31.08 | 49.41 | 99.97 |
|  | 14.69 | 19.12 | 46.08 | 19.00 | 41.81 | 70.10 |
| β-actin | 190.89 | 192.53 | 224.70 | 274.77 | 249.62 | 238.30 |
|  | 226.47 | 266.47 | 287.54 | 267.12 | 228.90 | 244.71 |
|  | 290.36 | 259.70 | 311.15 | 230.24 | 230.21 | 160.50 |

**3.Using mitochondrial autophagy inhibitors, such as Mdivi-1, autophagy inhibitor 3-MA, and lysosome degradation inhibitor Lys05, pre-treatments were conducted respectively. Then, glucose deprivation was carried out for 24 hours, and WB was used to detect the expression levels of FH and the reference gene; （Figure 2H）**

The concentration of mitochondrial autophagy inhibitor Mdivi-1 was 10 μM

The concentration of autophagy inhibitor 3-MA was 5 mM

Cell groups of HCT-116 and LS174T:

A cells: Glucose (+) + DMSO

B cells: Glucose (-) + DMSO

C cells: Glucose (-) + DMSO + Mdivi-1

D cells: Glucose (-) + DMSO + 3-MA

E cells: Glucose (-) + DMSO + Lys05

HCT-116	LS174T

FH

β-actin

|  | A | B | C | D | E | A | B | C | D | E |
| --- | --- | --- | --- | --- | --- | --- | --- | --- | --- | --- |
| FH | 58.41 | 24.17 | 25.62 | 54.53 | 51.24 | 71.63 | 27.46 | 26.40 | 70.21 | 74.82 |
|  | 45.36 | 15.90 | 17.31 | 40.02 | 46.04 | 68.31 | 24.00 | 25.19 | 63.89 | 71.69 |
|  | 101.65 | 28.83 | 31.02 | 119.71 | 114.41 | 43.38 | 21.00 | 15.62 | 50.25 | 55.51 |
| β-actin | 360.71 | 295.45 | 299.14 | 284.76 | 315.39 | 190.64 | 165.52 | 174.66 | 203.76 | 163.98 |
|  | 339.44 | 324.59 | 337.94 | 319.53 | 314.01 | 265.76 | 255.46 | 259.21 | 237.31 | 239.56 |
|  | 260.55 | 267.72 | 262.92 | 259.35 | 262.74 | 199.81 | 219.41 | 220.79 | 195.98 | 194.82 |

**4.FH Degradation Pathway under Glucose Starvation （Figure2 F）**

Under glucose starvation conditions, the degradation of FH occurs primarily through the autophagy–lysosomal system.

Colorectal cancer cell lines HCT-116 and LS174T were cultured under either Glucose(+) conditions (5 mM glucose, normal culture) or Glucose(–) conditions (glucose starvation). Cells were treated with specific pathway inhibitors:

Lys05 (50 µM, a dimeric chloroquine) to inhibit the autophagy–lysosomal pathway,

MG132 (10 µM) to inhibit the ubiquitin–proteasome pathway, and

Z-VAD-FMK (20 µM) to inhibit the caspase-dependent proteolytic pathway.

Results showed that inhibition of the autophagy–lysosomal pathway by Lys05 effectively restored FH protein levels, enhanced ferroptosis, and reduced cell viability and invasive capacity, indicating that cancer cells became less tolerant to glucose starvation. In contrast, the normal Glucose(+) group exhibited no significant changes.

Grouping of HCT-116 and LS174T cells:

A1: Glucose(+) + DMSO — control (normal culture)

A2: Glucose(+) + Lys05 (50 µM) — inhibition of autophagy–lysosomal pathway

A3: Glucose(+) + MG132 (10 µM) — inhibition of ubiquitin–proteasome pathway

A4: Glucose(+) + Z-VAD-FMK (20 µM) — inhibition of caspase-dependent pathway

B1: Glucose(–) + DMSO — glucose starvation control

B2: Glucose(–) + Lys05 (50 µM) — inhibition of autophagy–lysosomal pathway

B3: Glucose(–) + MG132 (10 µM) — inhibition of ubiquitin–proteasome pathway

B4: Glucose(–) + Z-VAD-FMK (20 µM) — inhibition of caspase-dependent pathway

HCT-116	LS174T

 FH

 FH

 FH

β-actin

β-actin

β-actin

|  | HCT-116 | | | | | | | | LS174T | | | | | | | |
| --- | --- | --- | --- | --- | --- | --- | --- | --- | --- | --- | --- | --- | --- | --- | --- | --- |
|  | A1 | A2 | A3 | A4 | B1 | B2 | B3 | B4 | A1 | A2 | A3 | A4 | B1 | B2 | B3 | B4 |
| FH | 80.85 | 87.69 | 74.51 | 85.13 | 22.20 | 67.20 | 22.00 | 23.03 | 122.76 | 110.69 | 98.09 | 121.44 | 20.93 | 117.05 | 24.03 | 37.35 |
|  | 125.12 | 106.58 | 120.22 | 133.21 | 28.67 | 188.20 | 17.88 | 21.69 | 148.71 | 145.81 | 191.09 | 183.50 | 17.67 | 156.15 | 35.46 | 38.82 |
|  | 121.80 | 121.10 | 136.76 | 160.88 | 23.08 | 131.07 | 26.49 | 23.26 | 193.56 | 232.27 | 201.79 | 229.42 | 32.36 | 225.38 | 30.51 | 37.08 |
| β-actin | 181.94 | 191.82 | 237.36 | 204.63 | 212.77 | 260.38 | 206.02 | 204.39 | 254.87 | 184.98 | 236.14 | 220.07 | 238.61 | 220.59 | 256.29 | 244.71 |
|  | 141.19 | 150.86 | 158.16 | 174.54 | 159.24 | 156.01 | 153.88 | 171.09 | 146.13 | 180.13 | 175.07 | 182.35 | 190.51 | 202.86 | 145.24 | 166.34 |
|  | 168.28 | 163.46 | 148.62 | 164.98 | 185.11 | 192.42 | 183.49 | 163.79 | 151.93 | 162.05 | 159.98 | 215.04 | 187.20 | 170.45 | 152.93 | 158.34 |

**4.Protein Stability Assay （Figure2 E）**

HCT-116 and LS174T colorectal cancer cells were divided into the following groups:

A1: Glucose(+) + DMSO — control (normal culture group)

A2: Glucose(+) + Lys05 (50 µM) — inhibition of lysosomal pathway

B1: Glucose(–) + DMSO — glucose starvation control group

B2: Glucose(–) + Lys05 (50 µM) — inhibition of lysosomal pathway

Cells were subjected to glucose starvation and Lys05 treatment for 24 hours.

At 0, 2, 4, 8, and 16 hours before sample collection, the protein synthesis inhibitor cycloheximide (CHX, 10 µg/mL) was added to block new protein synthesis.

Western blotting (WB) was performed to detect FH protein expression, using internal control proteins as loading references.

All experiments were independently repeated three times.

**HCT-116**

FH	β-actin	FH	β-actin

Times(h)	0 2 4 8 16	0 2 4 8 16	0 2 4 8 16	0 2 4 8 16

A1 A2

B1 B2

A1 A2

B1 B2

A1 A2

B1 B2

**LS174T**

FH	β-actin	FH	β-actin

Times(h)	0 2 4 8 16	0 2 4 8 16	0 2 4 8 16	0 2 4 8 16

A1 A2

B1 B2

A1 A2

B1 B2

A1 A2

B1 B2

|  |  | FH | | | | | β-actin | | | | |
| --- | --- | --- | --- | --- | --- | --- | --- | --- | --- | --- | --- |
| Times(h) |  | 0 | 2 | 4 | 8 | 16 | 0 | 2 | 4 | 8 | 16 |
| HCT-116 | A1 | 163.88 | 87.69 | 64.97 | 62.10 | 50.96 | 383.41 | 382.96 | 295.60 | 320.53 | 346.70 |
|  | A2 | 148.37 | 91.50 | 76.08 | 71.81 | 33.97 | 252.00 | 214.67 | 230.43 | 194.67 | 243.11 |
|  | B1 | 75.59 | 18.59 | 8.40 | 0.00 | 0.00 | 380.71 | 343.57 | 337.93 | 302.30 | 305.69 |
|  | B2 | 180.40 | 145.84 | 118.47 | 95.93 | 65.95 | 189.21 | 236.41 | 244.80 | 310.86 | 218.74 |
|  | A1-2 | 332.06 | 212.91 | 139.27 | 71.40 | 69.66 | 275.33 | 306.28 | 267.53 | 215.04 | 230.42 |
|  | A2-2 | 295.63 | 159.55 | 153.82 | 65.35 | 79.24 | 309.24 | 234.80 | 265.95 | 182.31 | 201.47 |
|  | B1-2 | 92.42 | 24.77 | 15.92 | 0.00 | 0.00 | 358.00 | 440.92 | 414.67 | 291.29 | 366.16 |
|  | B2-2 | 461.44 | 311.91 | 192.64 | 115.04 | 125.56 | 215.40 | 270.69 | 259.46 | 225.47 | 247.73 |
|  | A1-3 | 223.63 | 175.35 | 125.12 | 70.33 | 45.87 | 318.77 | 339.90 | 275.16 | 262.41 | 300.90 |
|  | A2-3 | 255.31 | 103.88 | 90.37 | 65.38 | 48.99 | 202.63 | 225.23 | 249.09 | 186.27 | 149.74 |
|  | B1-3 | 61.64 | 18.81 | 10.93 | 8.10 | 2.36 | 309.32 | 201.37 | 276.20 | 253.46 | 236.55 |
|  | B2-3 | 405.15 | 167.06 | 103.99 | 94.75 | 85.62 | 259.48 | 326.19 | 247.05 | 235.56 | 234.91 |
| LS174T | A1 | 257.16 | 191.44 | 138.98 | 121.00 | 109.59 | 209.54 | 172.31 | 160.90 | 224.23 | 286.70 |
|  | A2 | 280.56 | 157.18 | 136.37 | 109.68 | 95.74 | 253.28 | 322.09 | 279.90 | 302.65 | 218.57 |
|  | B1 | 54.24 | 15.54 | 20.09 | 7.62 | 0.00 | 351.14 | 317.55 | 322.13 | 262.38 | 309.52 |
|  | B2 | 357.33 | 271.09 | 185.99 | 170.24 | 102.59 | 307.02 | 267.39 | 222.42 | 288.01 | 246.75 |
|  | A1-2 | 191.56 | 94.07 | 93.66 | 70.76 | 41.64 | 316.11 | 386.86 | 387.77 | 310.39 | 371.05 |
|  | A2-2 | 217.60 | 83.68 | 75.19 | 63.62 | 54.07 | 312.81 | 307.20 | 350.52 | 292.85 | 213.93 |
|  | B1-2 | 73.38 | 21.66 | 20.22 | 12.69 | 0.00 | 411.96 | 403.55 | 396.49 | 314.04 | 347.54 |
|  | B2-2 | 313.96 | 196.45 | 120.97 | 74.66 | 85.43 | 223.14 | 179.40 | 204.23 | 209.74 | 188.94 |
|  | A1-3 | 236.76 | 183.46 | 100.70 | 78.65 | 41.74 | 232.74 | 214.26 | 258.94 | 320.70 | 178.64 |
|  | A2-3 | 212.36 | 153.70 | 84.24 | 74.72 | 56.36 | 229.24 | 157.04 | 154.33 | 143.22 | 148.03 |
|  | B1-3 | 73.47 | 31.66 | 20.20 | 7.76 | 8.92 | 366.08 | 386.14 | 259.78 | 336.37 | 256.28 |
|  | B2-3 | 282.04 | 231.82 | 152.12 | 85.16 | 56.58 | 472.60 | 330.55 | 252.77 | 258.32 | 251.20 |

**5.Two types of specific markers for different types of cell death (apoptosis, necrosis, pyroptosis, autophagy) were detected in two HCT116 and LS174T colorectal cancer cells under different glucose concentrations (0 mM, 1 mM, 5 mM). （Figure3 A）**

Grouping of HCT116 and LS174T cells

Group A: Cells + GLU (+, 5 mM) normal glucose group

Group B: Cells + GLU (+, 1 mM) low glucose group

Group C: Cells + GLU (-) glucose deprivation group

Detection time: 0 h, 12 h, 24 h, 48 h, 72 h

HCT-116	LS174T

Bcl-2

caspase-3

beclin-1

p62/SQSTM1

Caspase1

GSDMD

SCL7A11

GPX4

β-actin

|  |  | HCT-116 | | | LS174T | | |
| --- | --- | --- | --- | --- | --- | --- | --- |
|  |  | A | B | C | A | B | C |
| Bcl-2 |  | 120.05 | 108.71 | 111.30 | 85.71 | 90.96 | 96.10 |
|  |  | 119.16 | 125.84 | 122.16 | 71.65 | 68.12 | 71.48 |
|  |  | 134.07 | 135.08 | 122.79 | 54.31 | 57.15 | 62.57 |
| caspase-3 |  | 31.28 | 28.25 | 28.63 | 35.22 | 37.64 | 38.47 |
|  |  | 30.13 | 31.13 | 28.29 | 38.44 | 35.43 | 35.44 |
|  |  | 31.12 | 29.24 | 27.95 | 33.55 | 29.09 | 32.12 |
| beclin-1 |  | 23.50 | 28.82 | 80.90 | 46.74 | 48.44 | 93.07 |
|  |  | 23.90 | 23.79 | 55.79 | 25.08 | 27.05 | 99.63 |
|  |  | 23.77 | 27.39 | 56.68 | 41.56 | 45.70 | 94.53 |
| p62 |  | 17.06 | 27.73 | 47.28 | 33.75 | 34.94 | 71.89 |
|  |  | 28.61 | 27.32 | 70.75 | 46.38 | 41.66 | 103.00 |
|  |  | 29.55 | 35.45 | 56.47 | 37.19 | 27.81 | 73.38 |
| Caspase 1 | 45 | 127.02 | 161.02 | 145.93 | 80.10 | 58.41 | 75.63 |
|  | 20 | 8.27 | 9.26 | 8.26 | 10.64 | 10.07 | 10.09 |
|  | 45 | 79.43 | 80.17 | 78.84 | 59.10 | 55.62 | 63.65 |
|  | 20 | 5.91 | 5.59 | 5.12 | 5.69 | 6.00 | 4.61 |
|  | 45 | 112.90 | 99.23 | 104.22 | 54.88 | 63.01 | 60.03 |
|  | 20 | 11.03 | 12.71 | 12.40 | 8.15 | 7.56 | 7.09 |
| GSDMD | 53 | 128.04 | 115.37 | 121.03 | 61.29 | 51.87 | 58.83 |
|  | 30 | 6.08 | 7.09 | 5.94 | 3.77 | 4.32 | 3.83 |
|  | 53 | 121.68 | 122.87 | 108.57 | 125.17 | 85.49 | 97.62 |
|  | 30 | 16.83 | 18.12 | 15.46 | 12.84 | 13.38 | 14.36 |
|  | 53 | 100.64 | 113.05 | 94.79 | 72.35 | 58.18 | 74.02 |
|  | 30 | 16.73 | 22.81 | 17.82 | 17.56 | 14.08 | 14.47 |
| SCL7A11 |  | 52.13 | 57.01 | 59.06 | 41.63 | 45.42 | 40.41 |
|  |  | 35.29 | 37.30 | 34.15 | 29.49 | 29.51 | 25.66 |
|  |  | 64.62 | 70.70 | 78.22 | 41.21 | 39.94 | 41.44 |
| GPX4 |  | 44.82 | 44.27 | 119.94 | 85.55 | 78.18 | 212.71 |
|  |  | 51.38 | 66.01 | 138.46 | 60.67 | 67.87 | 133.40 |

**6.Figure 3 Supplementary Detection （Figure 3B）**

Group A cells + GLU (+, 5 mM) Normal Glucose Group

Group B cells + GLU (-) Glucose Deprivation Group

Group C cells + GLU (-) + FH (OE) Glucose Deprivation Group

WB Detection of Apoptosis Markers: Anti-apoptotic protein Bcl-2, Pro-apoptotic cleaved caspase-3 (17/19 kDa, total molecular weight 32KD)

WB Detection of Autophagy Markers: beclin-1, p62 (SQSTM1), where the accumulation/upregulation of beclin-1 and p62 indicates autophagy inhibition, and the decrease of p62 indicates enhanced autophagy

WB Detection of Pyroptosis Markers: Caspase 1, GSDMD

WB Detection of Ferroptosis Markers: SCL7A11, GPX4

HCT-116	LS174T

FH

Bcl-2

caspase-3

beclin-1

p62/SQSTM1

Caspase1

GSDMD

SCL7A11

GPX4

β-actin

|  |  | HCT-116 | | | LS174T | | |
| --- | --- | --- | --- | --- | --- | --- | --- |
|  |  | A | B | C | A | B | C |
| FH |  | 91.63 | 59.82 | 193.17 | 104.51 | 58.92 | 261.37 |
|  |  | 73.06 | 42.58 | 176.76 | 123.56 | 41.21 | 295.94 |
|  |  | 70.07 | 43.91 | 228.90 | 85.40 | 27.33 | 269.63 |
| Bcl-2 |  | 154.18 | 140.50 | 136.54 | 100.52 | 109.91 | 108.84 |
|  |  | 171.53 | 185.06 | 200.71 | 96.52 | 87.50 | 100.00 |
|  |  | 174.39 | 163.29 | 182.89 | 117.63 | 104.63 | 99.34 |
| caspase-3 |  | 28.91 | 24.54 | 23.97 | 47.23 | 43.90 | 41.39 |
|  |  | 42.73 | 40.30 | 45.96 | 71.56 | 67.00 | 66.65 |
|  |  | 38.75 | 40.17 | 42.24 | 85.52 | 93.16 | 78.86 |
| beclin-1 |  | 68.65 | 196.79 | 209.31 | 113.71 | 237.94 | 244.09 |
|  |  | 54.06 | 271.91 | 293.50 | 133.27 | 264.32 | 290.26 |
|  |  | 56.05 | 272.03 | 305.93 | 112.57 | 283.31 | 253.61 |
| p62 |  | 36.24 | 105.66 | 86.89 | 39.72 | 104.67 | 113.17 |
|  |  | 36.63 | 126.94 | 117.03 | 50.65 | 130.11 | 116.28 |
|  |  | 30.15 | 175.54 | 194.63 | 35.23 | 249.95 | 234.76 |
| Caspase1 | 45 | 190.91 | 189.38 | 185.65 | 77.32 | 98.35 | 109.06 |
|  | 20 | 22.21 | 21.38 | 20.36 | 10.20 | 10.50 | 11.39 |
|  | 45 | 169.55 | 177.85 | 202.28 | 81.28 | 89.65 | 85.80 |
|  | 20 | 21.51 | 18.04 | 18.91 | 13.73 | 11.73 | 13.43 |
|  | 45 | 137.53 | 162.29 | 129.44 | 50.41 | 68.80 | 66.05 |
|  | 20 | 13.36 | 12.34 | 11.86 | 6.81 | 7.08 | 6.35 |
| GSDMD | 53 | 162.39 | 158.95 | 148.69 | 132.93 | 134.45 | 126.60 |
|  | 30 | 26.05 | 24.04 | 23.78 | 13.72 | 12.67 | 15.00 |
|  | 53 | 196.85 | 225.84 | 219.72 | 166.78 | 184.06 | 202.11 |
|  | 30 | 18.96 | 17.19 | 16.85 | 8.95 | 10.84 | 10.20 |
|  | 53 | 276.11 | 232.05 | 199.09 | 98.17 | 138.96 | 125.32 |
|  | 30 | 15.47 | 13.92 | 19.25 | 7.34 | 8.66 | 6.92 |
| SCL7A11 |  | 77.58 | 83.39 | 84.60 | 59.08 | 61.33 | 56.92 |
|  |  | 44.28 | 41.98 | 43.26 | 31.95 | 30.42 | 29.63 |
|  |  | 62.02 | 74.04 | 60.05 | 32.88 | 32.96 | 29.21 |
| GPX4 |  | 79.82 | 152.95 | 27.29 | 110.74 | 176.00 | 28.72 |
|  |  | 88.40 | 223.21 | 20.39 | 133.37 | 274.65 | 28.96 |
|  |  | 57.96 | 154.21 | 30.01 | 68.82 | 306.06 | 35.93 |
| β-actin |  | 292.55 | 326.64 | 308.36 | 294.51 | 273.55 | 268.50 |
|  |  | 337.39 | 356.04 | 343.71 | 253.14 | 235.60 | 274.24 |
|  |  | 327.08 | 304.56 | 327.88 | 234.61 | 217.78 | 209.52 |

**7.Detect the levels of FH proteins in each group, as well as the levels of FH succinylated through co-immunoprecipitation (Co-IP)** **（Figure 3C）**

Grouping of HCT116 and LS174T cells

Group A cells with Glu (+) in normal glucose group at 0h

Group B cells with Glu (-) in glucose deprivation group at 0h

Group C cells with Glu (-) in glucose deprivation group at 12h

Group D cells with Glu (-) in glucose deprivation group at 24h

Group E cells with Glu (-) in glucose deprivation group at 48h

Group F cells with Glu (-) in glucose deprivation group at 72h

HCT-116	LS174T

FH

GPX4

β-actin

|  | HCT-116 | | | | | | LS174T | | | | | |
| --- | --- | --- | --- | --- | --- | --- | --- | --- | --- | --- | --- | --- |
|  | A | B | C | D | E | F | A | B | C | D | E | F |
| FH | 72.66 | 74.23 | 34.34 | 8.88 | 9.17 | 8.41 | 79.61 | 72.91 | 19.55 | 6.43 | 5.17 | 5.45 |
|  | 115.33 | 97.20 | 66.61 | 17.81 | 11.78 | 12.55 | 75.42 | 85.07 | 42.43 | 8.20 | 6.52 | 5.48 |
|  | 47.61 | 53.18 | 19.82 | 6.38 | 4.90 | 5.41 | 39.84 | 66.32 | 41.32 | 7.19 | 5.53 | 4.65 |
| GPX4 | 76.66 | 85.31 | 207.51 | 336.93 | 319.62 | 286.21 | 68.78 | 70.34 | 199.55 | 309.28 | 321.34 | 296.46 |
|  | 55.36 | 52.63 | 83.55 | 230.77 | 237.87 | 258.30 | 108.63 | 105.37 | 275.09 | 374.07 | 362.45 | 398.71 |
|  | 80.38 | 104.90 | 171.47 | 320.95 | 328.51 | 272.04 | 102.07 | 76.09 | 334.69 | 483.29 | 493.20 | 450.72 |
| β-actin | 437.95 | 429.52 | 445.57 | 423.20 | 402.56 | 403.07 | 282.37 | 292.90 | 281.01 | 309.02 | 297.80 | 300.25 |
|  | 348.80 | 344.19 | 344.71 | 335.07 | 334.25 | 340.76 | 298.20 | 291.10 | 282.49 | 255.64 | 265.49 | 267.32 |
|  | 257.74 | 266.29 | 256.17 | 291.20 | 259.46 | 243.14 | 177.31 | 172.42 | 185.56 | 193.09 | 183.39 | 215.70 |

**8.Restoration of FH Protein Promotes Ferroptosis under Glucose Starvation Conditions（Figure3G）**

Restoration of FH protein levels effectively activated ferroptosis under glucose starvation conditions.

HCT-116 and LS174T colorectal cancer cells were divided into the following groups:

A1: Glucose(+) + DMSO — control (normal culture group)

A2: Glucose(+) + Lys05 (50 µM) — inhibition of lysosomal pathway

B1: Glucose(–) + DMSO — glucose starvation control group

B2: Glucose(–) + Lys05 (50 µM) — inhibition of lysosomal pathway

Each group was analyzed for the following indicators:

Western blot (WB) was used to detect FH expression and ferroptosis marker proteins, including glutathione peroxidase 4 (GPX4) and solute carrier family 7 member 11 (SLC7A11, also known as xCT).

SLC7A11

GXP4

FH

β-actin

|  | HCT-116 |  |  |  |  | LS174T |  |  |
| --- | --- | --- | --- | --- | --- | --- | --- | --- |
|  | A | B | C |  | A | B | C |  |
| SLC7A11 | 36.97 | 39.97 | 39.64 | 47.42 | 30.34 | 25.05 | 27.73 | 23.65 |
|  | 51.99 | 49.48 | 48.00 | 44.54 | 29.12 | 26.19 | 32.53 | 30.48 |
|  | 46.28 | 48.84 | 47.48 | 43.49 | 34.12 | 40.48 | 39.64 | 44.21 |
| GXP4 | 106.65 | 119.38 | 235.97 | 43.24 | 157.91 | 110.53 | 326.61 | 52.47 |
|  | 110.81 | 119.21 | 303.28 | 54.15 | 120.50 | 142.65 | 293.12 | 66.87 |
|  | 99.79 | 108.45 | 179.91 | 41.43 | 207.89 | 267.82 | 413.81 | 68.90 |
| FH | 96.40 | 86.62 | 35.64 | 106.18 | 83.23 | 102.98 | 53.35 | 96.44 |
|  | 52.57 | 81.94 | 50.11 | 108.26 | 74.75 | 74.50 | 50.21 | 126.83 |
|  | 88.31 | 68.19 | 33.73 | 73.92 | 76.94 | 83.96 | 51.04 | 90.03 |
| β-actin | 443.75 | 507.96 | 457.29 | 415.02 | 381.71 | 394.91 | 394.37 | 366.11 |
|  | 385.41 | 362.83 | 349.15 | 357.63 | 371.24 | 292.70 | 334.59 | 360.28 |
|  | 316.74 | 452.10 | 304.55 | 379.78 | 254.54 | 356.45 | 232.45 | 279.66 |

**9.Figure 3 L**

The experiment was divided into three groups with four mice per group. In total, eight mice were subcutaneously injected with HCT116-Lv-FH(OE) cells, and the remaining four mice were injected with HCT116-Lv-Vector cells. After tumors became macroscopically visible, the eight HCT116-Lv-FH(OE) tumor‑bearing mice were randomly divided into two subgroups (n = 4 for each group). One subgroup received local subcutaneous injection of Fer‑1 at the tumor site at a dose of 5 mg/kg body weight every other day (PMID: 22632970), while the other subgroup was treated with normal saline as a control. Tumor size was measured daily using a vernier caliper, and tumor volume was calculated by the formula: ((length \times width^2)/2). All mice were euthanized on day 25, and tumor tissues were collected for pathological examination and immunohistochemical analysis.

A HCT-116/NC-Vector

B HCT-116/FH(OE)

C HCT-116/FH(OE)+Fer-1

GPX4

FH

β-actin

|  | A |  |  |  | B |  |  |  | C |  |  |  |
| --- | --- | --- | --- | --- | --- | --- | --- | --- | --- | --- | --- | --- |
|  | 1 | 2 | 3 | 4 | 1 | 2 | 3 | 4 | 1 | 2 | 3 | 4 |
| GPX4 | 62.53 | 109.15 | 59.74 | 77.59 | 33.08 | 13.67 | 72.53 | 52.51 | 214.54 | 230.37 | 268.30 | 111.36 |
| FH | 37.91 | 20.05 | 75.59 | 35.76 | 141.65 | 98.68 | 189.87 | 158.01 | 124.96 | 123.94 | 133.94 | 187.20 |
| β-actin | 216.91 | 246.25 | 216.59 | 241.71 | 189.57 | 275.70 | 259.86 | 232.12 | 265.05 | 233.35 | 248.52 | 252.26 |

**10.Figure4G**

Group A: Glucose(+) + DMSO — normal culture group; cells grow normally with low ferroptosis level

Group B: Glucose(–) + DMSO — glucose starvation group; FH undergoes succinylation and degradation, leading to inhibition of ferroptosis

Group C: Glucose(–) + Lys05 — glucose starvation + FH restoration group; ferroptosis activation

Group D: Glucose(–) + Lys05 + DMF (40 µM) — fumarate intervention group; theoretically reverses the ferroptosis activation observed in Group C

Western blot (WB) was used to detect FH, GPX4, and ferritin heavy chain (FTH1) protein expression levels.

FH

GXP4

FTH1

β-actin

|  | HCT-116 | | | | LS174T | | | |
| --- | --- | --- | --- | --- | --- | --- | --- | --- |
|  | A | B | C | D | A | B | C | D |
| FH | 74.21 | 16.98 | 66.28 | 60.34 | 80.85 | 35.54 | 75.20 | 74.20 |
|  | 47.43 | 20.46 | 44.82 | 37.49 | 74.81 | 39.28 | 59.36 | 87.29 |
|  | 84.38 | 20.75 | 91.09 | 89.65 | 84.80 | 34.85 | 85.97 | 93.02 |
| GXP4 | 55.87 | 110.63 | 10.57 | 152.38 | 136.80 | 367.62 | 37.36 | 310.69 |
|  | 69.18 | 194.13 | 18.55 | 137.15 | 120.69 | 327.88 | 35.98 | 292.10 |
|  | 50.28 | 167.11 | 20.97 | 184.73 | 144.11 | 332.41 | 29.57 | 245.47 |
| FTH1 | 67.21 | 72.69 | 38.09 | 133.00 | 69.68 | 81.80 | 45.63 | 175.89 |
|  | 89.81 | 124.36 | 45.79 | 195.55 | 106.75 | 104.09 | 48.28 | 197.05 |
|  | 123.19 | 122.87 | 68.92 | 160.49 | 92.32 | 102.91 | 38.81 | 162.07 |
| β-actin | 226.67 | 249.91 | 222.60 | 201.53 | 181.10 | 209.57 | 186.50 | 188.79 |
|  | 229.99 | 249.58 | 242.92 | 248.48 | 188.01 | 169.37 | 167.17 | 179.43 |
|  | 210.50 | 210.73 | 183.33 | 222.08 | 232.90 | 201.92 | 186.90 | 163.82 |

**11.Cellular Thermal Shift Assay Figure5 C**

A +DMSO

B +[Fumarate](https://pubchem.ncbi.nlm.nih.gov/compound/5460307)

C +[ML385](https://www.selleck.cn/products/ml385-nrf2-inhibitor.html)

A	B	C

NRF2

β-actin

|  | Temp(℃) | 37 | 42 | 47 | 52 | 57 | 62 | 67 |
| --- | --- | --- | --- | --- | --- | --- | --- | --- |
| NRF2 | A | 166.62 | 152.62 | 94.61 | 33.78 | 22.18 | 5.21 | 166.62 |
|  | B | 217.17 | 247.38 | 239.71 | 192.08 | 47.24 | 15.14 | 217.17 |
|  | C | 190.00 | 206.56 | 213.12 | 160.49 | 53.56 | 18.96 | 190.00 |
| β-actin | A | 484.22 | 546.07 | 528.24 | 412.48 | 123.74 | 29.90 | 484.22 |
|  | B | 442.31 | 434.79 | 404.06 | 369.12 | 130.55 | 37.88 | 442.31 |
|  | C | 166.62 | 152.62 | 94.61 | 33.78 | 22.18 | 5.21 | 166.62 |

**12.NRF2 protein stability experiment Figure5F**

A+DMSO

B+[Fumarate](https://pubchem.ncbi.nlm.nih.gov/compound/5460307) (DMF ,10mΜ)

NRF2	NRF2	β-actin	β-actin

Times(h) 0 2 4 8 16	0 2 4 8 16	0 2 4 8 16 0 2 4 8 16

A BAB

AB	AB

ABAB

|  | NRF2 | | | | | β-actin | | | | |
| --- | --- | --- | --- | --- | --- | --- | --- | --- | --- | --- |
| Times(h) | 0 | 2 | 4 | 8 | 16 | 0 | 2 | 4 | 8 | 16 |
| A | 137.39 | 76.18 | 26.95 | 15.96 | 8.30 | 353.55 | 409.80 | 377.33 | 375.50 | 334.50 |
| B | 172.15 | 106.97 | 77.79 | 39.40 | 26.71 | 262.11 | 292.19 | 300.79 | 314.46 | 286.71 |
| A-2 | 183.60 | 104.40 | 33.60 | 15.58 | 9.44 | 374.30 | 369.18 | 384.79 | 382.65 | 342.82 |
| B-2 | 145.73 | 142.80 | 65.69 | 44.00 | 28.00 | 331.20 | 332.24 | 321.41 | 268.93 | 277.28 |
| A-3 | 127.73 | 86.51 | 38.83 | 19.21 | 10.26 | 410.53 | 383.25 | 427.44 | 406.43 | 439.31 |
| B-3 | 183.64 | 196.38 | 135.69 | 60.82 | 30.58 | 353.49 | 334.54 | 318.44 | 346.46 | 333.25 |

**13.Detect the changes in the interaction between KEAP1 and NRF2 as well as the modifications of NRF2 ubiquitination. Figure5G**

A Glucose(-)+DMSO

B Glucose(-)+[Fumarate](https://pubchem.ncbi.nlm.nih.gov/compound/5460307) (DMF ,5mΜ)

C Glucose(-)+[Fumarate](https://pubchem.ncbi.nlm.nih.gov/compound/5460307) (DMF ,10mΜ)

HCT-116	LS174T

IP:	IgG	NRF2	IgG	NRF2

IB-NRF2

IB-Ubiquitin

IB-KEAP1

Input-NRF2	Input-KEAP1

Input-β-actin

|  | IgG-A | IgG-B | IgG-C | A | B | C | IgG-A | IgG-B | IgG-C | A | B | C |
| --- | --- | --- | --- | --- | --- | --- | --- | --- | --- | --- | --- | --- |
| IP-NRF2  IB-NRF2 | 0.00 | 0.00 | 0.00 | 63.71 | 56.81 | 55.53 | 0.00 | 0.00 | 0.00 | 142.98 | 157.53 | 135.56 |
| IP-NRF2  IB-Ubiquitin | 0.00 | 0.00 | 0.00 | 192.88 | 75.65 | 18.01 | 0.00 | 0.00 | 0.00 | 345.05 | 140.29 | 50.93 |
| IP-NRF2  IB-KEAP1 | 0.00 | 0.00 | 0.00 | 63.75 | 23.62 | 7.68 | 0.00 | 0.00 | 0.00 | 51.27 | 29.05 | 8.39 |
| Input-NRF2 | ／ | | | 54.21 | 58.03 | 56.84 |  |  | ／ | 61.70 | 61.63 | 57.34 |
| Input-KEAP1 |  |  |  | 34.28 | 39.80 | 39.07 |  |  |  | 38.38 | 35.80 | 38.10 |
| Input-β-actin |  |  |  | 242.12 | 247.14 | 207.58 |  |  |  | 178.84 | 195.29 | 184.01 |

**14.WB was used to detect the expressions of NRF2, GPX4, and FTH1, and internal control detection was also conducted. Figure5 E**

HCT-116 and LS174T cell groups:

A: Glucose(-) + DMSO

B: Glucose(-) + Fumarate (DMF, 5 mM)

C: Glucose(-) + Fumarate (DMF, 10 mM)

HCT-116	LS174T	HCT-116	LS174T	HCT-116	LS174T

NRF2

GPX4	FTH1

FTH1

β-actin

|  | HCT-116 | | | LS174T | | |
| --- | --- | --- | --- | --- | --- | --- |
|  | A | B | C | A | B | C |
| NRF2 | 39.70 | 88.47 | 145.13 | 69.81 | 117.59 | 248.32 |
|  | 51.30 | 105.02 | 155.87 | 78.32 | 144.45 | 234.37 |
|  | 36.02 | 95.57 | 206.03 | 74.63 | 100.44 | 318.32 |
| GPX4 | 28.41 | 51.45 | 145.78 | 62.18 | 138.36 | 184.97 |
|  | 38.31 | 72.86 | 197.42 | 82.10 | 114.88 | 170.94 |
|  | 45.23 | 82.09 | 189.25 | 106.04 | 157.64 | 224.96 |
| FTH1 | 10.55 | 29.30 | 47.78 | 37.43 | 63.96 | 87.19 |
|  | 16.13 | 48.34 | 106.07 | 30.26 | 56.72 | 112.14 |
|  | 24.69 | 41.05 | 89.46 | 42.77 | 78.13 | 103.96 |
| β-actin | 270.34 | 291.72 | 321.56 | 302.05 | 325.00 | 293.12 |
|  | 378.74 | 339.50 | 350.03 | 299.75 | 301.49 | 280.72 |
|  | 266.92 | 207.61 | 245.44 | 255.78 | 234.13 | 229.39 |

**15.Under glucose starvation conditions, ML385 reactivates ferroptosis inhibited by fumarate.**

**HCT-116：Figure5K**

A Glucose(+)+DMSO

B Glucose(-)+DMSO

C Glucose(-)+Lys05

D Glucose(-)+Lys05+DMF

E Glucose(-)+Lys05+DMF+[ML385](https://www.selleck.cn/products/ml385-nrf2-inhibitor.html)

FGlucose(-)+[ML385](https://www.selleck.cn/products/ml385-nrf2-inhibitor.html)

FH	NRF2

**FH**

NRF2

FTH1

GPX4	β-actin

**GPX4**

β-actin

|  | A | B | C | D | E | F |
| --- | --- | --- | --- | --- | --- | --- |
| FH | 29.02 | 8.96 | 140.62 | 44.52 | 46.49 | 202.56 |
|  | 31.03 | 7.39 | 75.58 | 26.31 | 23.90 | 80.51 |
|  | 52.24 | 19.97 | 122.93 | 46.36 | 47.95 | 156.05 |
| NRF2 | 101.66 | 220.55 | 34.75 | 169.49 | 29.05 | 27.61 |
|  | 65.49 | 155.53 | 16.57 | 146.61 | 17.56 | 13.74 |
|  | 42.68 | 173.49 | 17.92 | 127.63 | 12.04 | 12.29 |
| FTH1 | 22.53 | 55.93 | 7.24 | 39.84 | 9.47 | 9.71 |
|  | 18.85 | 82.09 | 7.97 | 47.43 | 5.45 | 5.77 |
|  | 38.86 | 71.53 | 9.37 | 61.01 | 10.00 | 9.54 |
| GPX4 | 64.12 | 295.80 | 22.14 | 222.64 | 22.45 | 16.40 |
|  | 56.16 | 258.64 | 15.14 | 171.43 | 10.59 | 7.68 |
|  | 79.21 | 307.50 | 27.36 | 260.38 | 33.62 | 24.42 |
| β-actin | 249.29 | 291.88 | 224.80 | 321.95 | 262.17 | 274.20 |
|  | 306.62 | 334.26 | 320.07 | 356.53 | 362.53 | 276.99 |
|  | 338.20 | 341.80 | 327.51 | 347.72 | 298.81 | 253.23 |

**16.Figure6 B**

HCT-116	LS174T

IB: Succ

IB: FH

Input:β-actin

|  | HCT-116 | | | | | | LS174T | | | | | |
| --- | --- | --- | --- | --- | --- | --- | --- | --- | --- | --- | --- | --- |
|  | A | B | C | D | E | F | A | B | C | D | E | F |
| IP:FH IB: Succ | 22.31 | 23.26 | 70.35 | 270.51 | 226.52 | 271.19 | 34.01 | 36.91 | 117.71 | 294.15 | 321.89 | 304.48 |
| IP: FH IB: FH | 70.14 | 78.61 | 42.29 | 32.94 | 26.21 | 31.30 | 67.43 | 87.00 | 49.67 | 22.10 | 23.26 | 22.20 |
| Input:β-actin | 453.80 | 431.39 | 422.23 | 466.07 | 453.34 | 451.51 | 347.51 | 352.20 | 349.33 | 322.34 | 342.78 | 352.55 |

**17.Figure6 C**

IP:FH	HCT-116	LS174T	HCT-116	LS174T

IB:Succ	IB:CPT1A

Input:FH	Input:CPT1A

Input:β-actin

|  | HCT-116 | | | LS174T | | |
| --- | --- | --- | --- | --- | --- | --- |
|  | A1 | A2 | B | A1 | A2 | B |
| IP:FH IB:Succ | 28.26 | 193.21 | 25.01 | 59.42 | 200.53 | 53.64 |
| IP:FH IB:CPT1A | 58.74 | 198.28 | 23.39 | 43.65 | 168.68 | 17.32 |
| Input:FH | 124.55 | 32.66 | 116.00 | 121.33 | 30.95 | 112.78 |
| Input:CPT1A | 106.13 | 237.91 | 9.26 | 91.39 | 195.68 | 8.30 |
| Input:β-actin | 335.85 | 316.32 | 325.24 | 342.69 | 352.39 | 305.33 |

**18.Figure 6E**

Genetic intervention experiments revealed that CPT1A knockdown restored the ferroptosis sensitivity of colorectal cancer cells under glucose starvation conditions.

Colorectal cancer cells were transfected to establish the CPT1A knockdown model, to investigate whether CPT1A silencing could reverse the reduction of FH protein expression induced by glucose starvation, and further facilitate ferroptosis：

A Glucose(+)+NC vector

B Glucose(+)+CPT1A(KD)

C Glucose(-)+NC vector

D Glucose(-)+CPT1A(KD)

HCT-116	LS174T	HCT-116	LS174T	HCT-116	LS174T

CPT1A

GPX4

FH

β-actin

|  | HCT-116 | | | | LS174T | | | |
| --- | --- | --- | --- | --- | --- | --- | --- | --- |
|  | A | B | C | D | A | B | C | D |
| CPT1A | 50.96 | 19.19 | 133.26 | 21.10 | 62.45 | 10.94 | 129.87 | 18.44 |
|  | 26.77 | 8.53 | 61.67 | 9.46 | 56.56 | 4.75 | 110.22 | 9.17 |
|  | 43.06 | 13.10 | 93.83 | 8.56 | 39.67 | 14.04 | 74.47 | 9.57 |
| GPX4 | 71.96 | 65.92 | 190.14 | 13.76 | 88.84 | 179.19 | 336.23 | 30.84 |
|  | 50.84 | 58.74 | 78.74 | 29.77 | 91.15 | 73.52 | 168.85 | 18.99 |
|  | 42.12 | 41.31 | 69.56 | 32.92 | 78.62 | 86.56 | 148.26 | 33.73 |
| FH | 65.38 | 59.47 | 31.87 | 80.68 | 97.07 | 67.14 | 26.56 | 63.02 |
|  | 49.81 | 57.99 | 22.73 | 75.12 | 56.09 | 49.08 | 26.74 | 79.47 |
|  | 69.20 | 61.92 | 26.60 | 84.51 | 52.03 | 57.37 | 15.11 | 65.49 |
| β-actin | 235.56 | 275.35 | 217.00 | 269.23 | 215.81 | 231.83 | 259.09 | 230.84 |
|  | 188.92 | 186.55 | 191.20 | 252.42 | 219.55 | 208.91 | 238.03 | 268.10 |
|  | 177.17 | 218.08 | 182.67 | 259.13 | 236.10 | 233.44 | 189.43 | 200.90 |

19. **Figure 6D**

To identify the modification sites of FH succinylation and explore its correlation with ferroptosis.

CPT1A was overexpressed in HCT-116 and LS174T cells. Cell lysates were subjected to immunoprecipitation (IP) using an anti-FH antibody. The immunoprecipitated complexes were analyzed by immunoblotting (IB) with anti-succinyllysine rabbit polyclonal antibody and anti-CPT1A antibody, respectively. An IgG group was set as the negative control. In the input samples, the protein levels of CPT1A, FH, and the internal reference β‑actin were detected by IB.

A vector NC

B CPT1A(OE)

HCT-116	LS174T	HCT-116	LS174T

IP:	IgG	FH	IgG	FH	IgG	FH	IgG	FH

A B A B	A B A B	A B A B	A B A B

IB:Succ	IB:CPT1A

HCT-116	LS174T	HCT-116 LS174T

Input:CPT1A	Input:FH

Input:β-actin

|  | HCT-116 | | | | LS174T | | | |
| --- | --- | --- | --- | --- | --- | --- | --- | --- |
|  | IgG | | FH | | IgG | | FH | |
|  | A | B | A | B | A | B | A | B |
| IP:FH IB:Succ | 0.00 | 0.00 | 60.24 | 225.05 | 0.00 | 0.00 | 89.39 | 210.72 |
| IP:FH IB:CPT1A | 0.00 | 0.00 | 26.84 | 157.93 | 0.00 | 0.00 | 88.94 | 215.05 |
| Input:CPT1A |  | | 52.56 | 283.15 |  | | 64.11 | 249.65 |
| Input:FH |  |  | 88.95 | 43.92 |  |  | 142.75 | 33.57 |
| Input:β-actin |  |  | 384.50 | 339.58 |  |  | 303.33 | 310.67 |

**20.Figure7 B**

Exogenous co‑immunoprecipitation (Co‑IP) assay was performed to identify the key sites of FH succinylation.

Based on the FH succinylation sites identified by protein modification omics, plasmids including FLAG‑CPT1A, wild‑type HA‑FH (WT), and three mutant HA‑FH constructs (K66R, K80R, K223R) were constructed. In these mutants, lysine (K) was mutated to arginine (R) to block succinylation modification. The above plasmids were transfected into two colorectal cancer cell lines. Cell lysates were immunoprecipitated with anti‑HA antibody, and the precipitated proteins were analyzed by immunoblotting using anti‑FLAG and anti‑succinyllysine antibodies. HA, FLAG and β‑actin protein levels in the input samples were simultaneously detected as controls.

| HCT-116, LS174T | 1 | 2 | 3 | 4 | 5 |
| --- | --- | --- | --- | --- | --- |
| HA-FH (WT) | — | + | — | — | — |
| HA -FH (K66R) | — | — | + | — | — |
| HA -FH (K80R) | — | — | — | + | — |
| HA -FH (K223R) | — | — | — | — | + |
| FLAG-CPT1A | + | + | + | + | + |

HCT-116

IB:FLAG	IB:Succ	IP:HA	Input:FLAG

Input:β-actin

LS174T：

IB:FLAG	IB:Succ

IP:HA	Input:FLAG

Input:β-actin

|  | HCT-116 | | | | | LS174T | | | | |
| --- | --- | --- | --- | --- | --- | --- | --- | --- | --- | --- |
|  | 1 | 2 | 3 | 4 | 5 | 1 | 2 | 3 | 4 | 5 |
| IP:HA IB:FLAG | 0.00 | 129.69 | 114.39 | 121.23 | 103.28 | 0.00 | 85.03 | 94.05 | 89.04 | 82.70 |
| IP:HA IB:Succ | 0.00 | 226.25 | 49.54 | 41.13 | 205.91 | 0.00 | 153.29 | 18.31 | 23.34 | 122.09 |
| Input:HA | 0.00 | 38.86 | 90.50 | 85.48 | 32.11 | 0.00 | 66.10 | 145.35 | 139.80 | 55.21 |
| Input:FLAG | 236.15 | 224.95 | 198.15 | 208.77 | 206.04 | 112.12 | 133.05 | 113.17 | 122.39 | 119.88 |
| Input:β-actin | 276.41 | 307.79 | 325.72 | 304.77 | 265.04 | 303.57 | 319.13 | 345.21 | 319.60 | 305.86 |

**21.Figure7 C**

Protein stability assay (detection of HA‑tagged protein) HA‑FH (wild‑type, WT), HA‑FH (K66R), HA‑FH (K80R) and HA‑FH (K66R/K80R) mutant plasmids were constructed. These plasmids were separately transfected into two colorectal cancer cell lines. Before cell collection, cells were treated with 10 μg/mL cycloheximide (CHX), a protein synthesis inhibitor, at 0, 2, 4, 8 and 16 h. Cells were harvested at the corresponding time points, and the expression levels of HA‑tagged FH protein were determined by Western blot, with β‑actin served as the internal reference control.

A HA-FH(WT),

B HA-FH(K66R),

C HA-FH(K80R)，

D HA-FH(K66R/K80R)

HCT-116

HA	β-actin	HA	β-actin

Times(h) 0 2 4 8 16	0 2 4 8 16	0 2 4 8 16	0 2 4 8 16

A B

C D

A B

C D

A B

C

D

LS174T

HA	β-actin	HA	β-actin

Times(h) 0 2 4 8 16	0 2 4 8 16	0 2 4 8 16 0 2 4 8 16

AB

CD

A	B

CD

A	B

CD

|  |  | HA | | | | | β-actin | | | | |
| --- | --- | --- | --- | --- | --- | --- | --- | --- | --- | --- | --- |
| Times(h) |  | 0 | 2 | 4 | 8 | 16 | 0 | 2 | 4 | 8 | 16 |
| HCT-116 | A | 110.94 | 40.12 | 14.80 | 5.33 | 0.00 | 406.05 | 382.83 | 369.28 | 380.06 | 348.53 |
|  | B | 120.26 | 86.78 | 65.66 | 51.30 | 35.34 | 466.38 | 470.20 | 435.87 | 399.92 | 428.54 |
|  | C | 162.65 | 135.87 | 51.15 | 24.36 | 16.22 | 417.61 | 394.32 | 405.84 | 362.73 | 388.40 |
|  | D | 144.25 | 129.13 | 97.15 | 92.94 | 100.75 | 452.38 | 441.21 | 382.32 | 397.47 | 353.24 |
|  | A-2 | 103.50 | 34.46 | 12.84 | 0.00 | 0.00 | 225.89 | 184.84 | 205.17 | 170.89 | 155.18 |
|  | B-2 | 121.19 | 87.13 | 61.12 | 53.46 | 22.38 | 250.40 | 213.85 | 207.00 | 173.49 | 184.13 |
|  | C-2 | 95.47 | 59.40 | 25.32 | 14.89 | 6.94 | 214.71 | 198.19 | 212.53 | 185.88 | 195.55 |
|  | D-2 | 85.50 | 96.42 | 57.86 | 70.67 | 73.77 | 193.83 | 223.06 | 212.51 | 207.29 | 195.69 |
|  | A-3 | 133.85 | 47.28 | 15.02 | 0.00 | 0.00 | 263.63 | 261.26 | 275.79 | 263.69 | 229.92 |
|  | B-3 | 256.40 | 186.31 | 123.73 | 61.91 | 48.00 | 361.16 | 351.71 | 347.82 | 323.53 | 331.73 |
|  | C-3 | 206.60 | 123.15 | 55.60 | 35.21 | 27.90 | 215.02 | 209.17 | 196.55 | 215.68 | 175.44 |
|  | D-3 | 235.76 | 243.56 | 215.69 | 215.26 | 187.88 | 286.42 | 270.24 | 298.93 | 246.44 | 226.16 |
| LS174T | A | 93.43 | 29.06 | 9.44 | 6.58 | 0.00 | 447.94 | 353.59 | 384.54 | 328.72 | 367.57 |
|  | B | 128.10 | 118.28 | 66.87 | 54.32 | 51.62 | 439.37 | 341.36 | 342.99 | 329.79 | 281.24 |
|  | C | 149.27 | 86.08 | 65.01 | 44.31 | 38.20 | 455.55 | 367.94 | 350.02 | 463.68 | 376.79 |
|  | D | 184.15 | 161.14 | 170.66 | 123.71 | 112.87 | 249.44 | 358.64 | 311.31 | 306.15 | 286.82 |
|  | A-2 | 122.33 | 35.26 | 22.46 | 4.50 | 0.00 | 214.86 | 224.62 | 183.12 | 164.33 | 199.36 |
|  | B-2 | 115.83 | 118.80 | 81.79 | 61.12 | 35.89 | 194.24 | 156.73 | 184.36 | 133.68 | 157.66 |
|  | C-2 | 119.03 | 156.76 | 43.49 | 24.43 | 10.37 | 254.52 | 233.37 | 271.35 | 257.64 | 235.79 |
|  | D-2 | 130.21 | 143.57 | 142.94 | 166.25 | 102.76 | 285.37 | 266.08 | 245.12 | 237.85 | 205.22 |
|  | A-3 | 165.99 | 26.17 | 9.68 | 0.00 | 0.00 | 197.09 | 198.24 | 155.87 | 149.91 | 143.15 |
|  | B-3 | 241.98 | 177.74 | 109.73 | 67.48 | 40.31 | 219.78 | 180.08 | 162.34 | 151.27 | 177.21 |
|  | C-3 | 226.29 | 185.61 | 93.76 | 17.99 | 15.85 | 179.70 | 196.99 | 211.01 | 160.98 | 179.20 |
|  | D-3 | 221.77 | 203.54 | 145.76 | 157.94 | 90.14 | 162.31 | 171.33 | 181.87 | 142.57 | 169.10 |

**22.Figure 7D**

**In vitro succinylation assay**

| Group | Group1 | Group2 | Group3 | Group4 | Group5 | Group6 |
| --- | --- | --- | --- | --- | --- | --- |
| Flag-FH(WT) | + | — | + |  | + |  |
| Flag-FH(K66R/K80R) | — | + | — | + | — | + |
| CPT1A | + | + | — | — | + | + |
| succinyl-CoA(1mmol/L) | — | — | + | + | + | + |

|  | Group1 | Group2 | Group3 | Group4 | Group5 | Group6 |
| --- | --- | --- | --- | --- | --- | --- |
| Succ | 0.00 | 0.00 | 9.03 | 0.00 | 132.68 | 7.93 |
|  | 0.00 | 0.00 | 10.55 | 0.00 | 58.56 | 8.52 |
|  | 0.00 | 0.00 | 8.96 | 0.00 | 175.47 | 9.55 |
| INPUT:Flag-FH | 187.13 | 177.49 | 187.21 | 181.15 | 191.05 | 174.88 |
|  | 137.61 | 160.46 | 158.96 | 160.95 | 166.92 | 148.02 |
|  | 144.93 | 153.99 | 143.93 | 141.98 | 144.35 | 145.21 |

**23.Figure 7 F**

Mutation of FH-K66/K80 restores the ferroptosis sensitivity of colorectal cancer cells under glucose starvation

Under glucose starvation, the FH-K66R/K80R double mutation blocks CPT1A-mediated FH succinylation, thereby reducing the autophagic‑lysosomal degradation of FH. The accumulation of intracellular FH protein elevates labile iron levels and lipid peroxidation, ultimately triggering ferroptosis and impairing the tolerance of colorectal cancer cells to glucose starvation, which exerts tumor‑suppressive effects. In contrast, wild-type FH is subjected to succinylation modification and subsequent degradation, resulting in insufficient intracellular FH abundance and only partial inhibition of ferroptosis.

To eliminate the interference of endogenous FH and ensure the efficiency of exogenous plasmid transfection, endogenous FH was firstly knocked out in this experiment.

A Glucose(+)+vector NC

B Glucose(+)+FH(KD)

C Glucose(-)+FH(KD)

D Glucose(-)+FH(KD)+FH(WT)

E Glucose(-)+FH(KD)+FH(K66R+K80R)

HCT-116	LS174T	HCT-116	LS174T

FTH1

GPX4

FH

β-actin

|  | HCT-116 | | | | | LS174T | | | | |
| --- | --- | --- | --- | --- | --- | --- | --- | --- | --- | --- |
|  | A | B | C | D | E | A | B | C | D | E |
| FTH1 | 75.73 | 152.73 | 107.27 | 41.00 | 16.13 | 103.74 | 212.90 | 274.53 | 51.38 | 11.85 |
| -2 | 38.97 | 185.62 | 130.39 | 26.15 | 13.17 | 79.27 | 194.63 | 141.31 | 41.05 | 9.00 |
| -3 | 64.10 | 154.55 | 139.09 | 37.98 | 15.15 | 92.05 | 270.32 | 199.20 | 51.36 | 18.02 |
| GPX4 | 44.24 | 96.61 | 253.51 | 23.49 | 9.96 | 126.27 | 186.93 | 222.47 | 34.11 | 10.20 |
| -2 | 115.13 | 281.51 | 365.74 | 44.85 | 21.47 | 173.46 | 404.86 | 398.38 | 66.38 | 21.48 |
| -3 | 61.04 | 229.36 | 128.21 | 24.84 | 8.09 | 134.02 | 338.22 | 347.28 | 39.72 | 6.13 |
| FH | 39.64 | 11.50 | 11.88 | 20.21 | 172.35 | 51.04 | 13.73 | 10.05 | 41.18 | 306.95 |
| -2 | 38.31 | 0.00 | 0.00 | 31.14 | 0.00 | 65.75 | 0.00 | 0.00 | 29.23 | 177.94 |
| -3 | 46.92 | 0.00 | 0.00 | 17.53 | 124.22 | 66.14 | 0.00 | 0.00 | 52.91 | 235.54 |
| β-actin | 289.66 | 320.72 | 295.07 | 248.31 | 243.73 | 267.75 | 258.86 | 276.25 | 300.61 | 272.32 |
| -2 | 379.47 | 320.55 | 321.93 | 347.91 | 322.33 | 315.97 | 286.13 | 339.07 | 195.88 | 201.73 |
| -3 | 275.55 | 322.42 | 322.16 | 291.81 | 276.83 | 206.06 | 234.53 | 285.73 | 300.18 | 274.07 |

**24.Figure 7 J**

**Using deferoxamine (DFO) for rescue experiments is necessary to confirm the cell death inhibited by the fumarate salts.**

A Glucose(-)+vector oncogenic group

B Glucose(-)+FH(WT) partially activates ferroptosis

C Glucose(-)+FH(K66R/K80R) significantly inhibits cancer group DGlucose(-)+FH(K66R/K80R)+Fer-1

E Glucose(-)+FH(K66R/K80R)+DFO

FH	FTH1

GPX4	β-actin

|  | A | B | C | D | E |
| --- | --- | --- | --- | --- | --- |
| FH | 12.63 | 36.91 | 118.59 | 133.68 | 126.48 |
|  | 11.09 | 25.91 | 73.84 | 105.55 | 117.08 |
|  | 10.39 | 33.10 | 107.43 | 109.30 | 175.47 |
| FTH1 | 49.79 | 24.70 | 11.91 | 140.74 | 155.01 |
|  | 34.96 | 20.38 | 7.44 | 129.59 | 140.21 |
|  | 57.38 | 27.76 | 10.90 | 144.02 | 149.04 |
| GPX4 | 79.51 | 27.40 | 9.86 | 153.40 | 202.48 |
|  | 96.71 | 34.09 | 13.25 | 137.33 | 153.71 |
|  | 87.84 | 34.89 | 13.04 | 277.21 | 283.46 |
| β-actin | 284.24 | 322.09 | 312.95 | 259.50 | 245.14 |
|  | 383.68 | 396.34 | 384.90 | 393.06 | 375.76 |
|  | 387.06 | 380.23 | 375.74 | 401.42 | 355.96 |

**25.Figure S1 A**

**Cell Screening Experiment:**

Western blot analysis was performed to detect FH protein levels in colorectal cancer cell lines (Caco-2, RKO, HT29,HCT-116, COLO205, T84, LS174T, and LS180). Based on the relative expression levels of FH, cell lines with lower FH protein expression were selected for subsequent experiments.

FH	β-actin

|  | Caco-2 | RKO | HT29 | HCT-116 | COLO205 | T84 | LS174T | LS180 |
| --- | --- | --- | --- | --- | --- | --- | --- | --- |
| FH | 25.84 | 166.53 | 165.93 | 78.79 | 138.78 | 18.74 | 89.35 | 26.79 |
|  | 28.80 | 208.15 | 179.94 | 115.61 | 181.32 | 16.14 | 93.98 | 22.64 |
|  | 21.80 | 205.00 | 186.09 | 85.60 | 180.92 | 34.30 | 95.17 | 32.90 |
| β-actin | 192.71 | 274.73 | 186.46 | 241.70 | 202.65 | 135.00 | 191.01 | 156.92 |
|  | 196.85 | 243.62 | 169.62 | 205.70 | 190.36 | 202.10 | 211.35 | 170.57 |
|  | 210.46 | 249.11 | 170.65 | 194.92 | 152.74 | 156.66 | 177.52 | 169.48 |

**26.Figure S2 D**

**Verify that fumaric acid can inhibit ferroptosis**

Group HCT116 and LS174T cells, under normal culture conditions

Group A cells + DMSO

Group B cells + succinic DES (10 nM)

HCT-116 LS174T

FH	GPX4

FH

GPX4	β-actin

β-actin

|  | HCT-116 | | LS174T | |
| --- | --- | --- | --- | --- |
|  | A | B | A | B |
| FH | 38.90 | 13.69 | 60.63 | 19.23 |
|  | 49.52 | 27.58 | 74.13 | 24.71 |
|  | 37.77 | 21.69 | 68.98 | 26.10 |
| GPX4 | 59.08 | 153.51 | 120.47 | 406.16 |
|  | 82.30 | 172.82 | 155.31 | 330.44 |
|  | 76.32 | 145.43 | 106.17 | 295.28 |
| β-actin | 425.12 | 436.91 | 316.86 | 324.56 |
|  | 263.84 | 282.94 | 241.71 | 225.93 |
|  | 349.49 | 374.55 | 250.10 | 273.15 |

**27.Figure S3 A**

Perform immunoprecipitation (IP) with anti-FH antibody, followed by immunoblotting (IB) to detect succinylation and KAT2A.

FH and KAT2A were measured in the input samples

IP:FH	HCT-116	LS174T	HCT-116	LS174T

IB:SuccIB:KAT2A

Input:FH	Input:KAT2A

Input:β-actin

|  | HCT-116 | | | LS174T | | |
| --- | --- | --- | --- | --- | --- | --- |
|  | A1 | A2 | C | A1 | A2 | C |
| IP:FH IB:Succ | 41.87 | 233.37 | 229.14 | 45.62 | 163.63 | 145.83 |
| IP:FH IB:KAT2A | 0.00 | 0.00 | 0.00 | 0.00 | 0.00 | 0.00 |
| Input:FH | 66.76 | 33.10 | 22.88 | 111.63 | 36.05 | 30.90 |
| Input:KAT2A | 108.50 | 92.17 | 16.52 | 178.05 | 164.51 | 14.06 |
| Input:β-actin | 221.28 | 227.02 | 181.71 | 156.07 | 184.92 | 164.09 |

Perform immunoprecipitation (IP) with anti-FH antibody, followed by immunoblotting (IB) to detect succinylation and EP300.

FH and HAT1 were detected in the input samples.

IP:FH	HCT-116	LS174T

IB:SuccIB:HAT1

Input:FH

Input:HAT1

Input:β-actin	Input:FH

|  | HCT-116 | | | LS174T | | |
| --- | --- | --- | --- | --- | --- | --- |
|  | A1 | A2 | D | A1 | A2 | D |
| IP:FH IB:Succ | 58.14 | 191.05 | 157.01 | 36.90 | 135.03 | 125.19 |
| IP:FH IB:HAT1 | 133.89 | 263.34 | 34.95 | 55.22 | 241.53 | 17.40 |
| Input:FH | 87.27 | 43.27 | 23.46 | 109.89 | 76.11 | 15.94 |
| Input:HAT1 | 144.49 | 143.04 | 19.16 | 95.98 | 79.51 | 14.88 |
| Input:β-actin | 226.31 | 179.32 | 205.98 | 181.73 | 179.50 | 169.14 |

Perform immunoprecipitation (IP) with anti-FH antibody, followed by immunoblotting (IB) to detect Succ and EP300.

FH and EP300 were measured in the input samples.

IP:FH	HCT-116	LS174T

IB:SuccIB:EP300

Input:FH	Input:EP300

Input:β-actin

|  | HCT-116 | | | LS174T | | |
| --- | --- | --- | --- | --- | --- | --- |
|  | A1 | A2 | E | A1 | A2 | E |
| IP:FH IB:Succ | 17.22 | 127.07 | 111.45 | 44.00 | 175.80 | 157.75 |
| IP:FH IB:EP300 | 66.99 | 195.38 | 0.00 | 83.16 | 240.66 | 0.00 |
| Input:FH | 125.66 | 60.49 | 0.00 | 52.04 | 30.20 | 0.00 |
| Input:EP300 | 49.20 | 58.20 | 0.00 | 104.74 | 88.03 | 0.00 |
| Input:β-actin | 279.22 | 292.47 | 327.38 | 183.36 | 211.33 | 210.68 |

**28.Figure S3 B**

Following intervention with the CPT1A inhibitor, co‑immunoprecipitation (Co‑IP) was performed to detect FH succinylation and CPT1A expression. Meanwhile, FH, CPT1A and the internal reference protein were examined in the input samples.

A Glucose(+)+DMSO

B Glucose(+)+Etomoxir

C Glucose(-)+DMSO

D Glucose(-)+Etomoxir

HCT-116	LS174T

IP:	IgG	FH	IgG	FH

A B C D	A B C D	A B C D	A B C D

IB:Succ

IB:CPT1A

Input:CPT1A

Input:FH

Input:β-actin

|  | HCT-116 | | | | | | | | LS174T | | | | | | | |
| --- | --- | --- | --- | --- | --- | --- | --- | --- | --- | --- | --- | --- | --- | --- | --- | --- |
|  | A | B | C | D | A | B | C | D | A | B | C | D | A | B | C | D |
| IP:FH IB:Succ | 0.00 | 0.00 | 0.00 | 0.00 | 42.02 | 51.64 | 96.20 | 47.27 | 0.00 | 0.00 | 0.00 | 0.00 | 43.41 | 48.89 | 155.28 | 38.11 |
| IP:FH IB:CPT1A | 0.00 | 0.00 | 0.00 | 0.00 | 24.99 | 28.95 | 97.56 | 92.65 | 0.00 | 0.00 | 0.00 | 0.00 | 44.65 | 43.10 | 99.82 | 94.77 |
| Input:CPT1A |  | | | | 30.23 | 32.74 | 73.63 | 88.97 |  | | | | 28.19 | 27.98 | 71.36 | 78.59 |
| Input:FH |  |  |  |  | 70.41 | 84.21 | 31.89 | 124.23 |  |  |  |  | 76.01 | 87.37 | 38.70 | 196.65 |
| Input:β-actin |  |  |  |  | 200.76 | 179.83 | 200.89 | 222.06 |  |  |  |  | 166.93 | 142.29 | 149.68 | 134.77 |

**29.Figure S3 C**

Colorectal cancer cells under glucose starvation were treated with etomoxir sodium salt, a specific CPT1A inhibitor (CPT1A acts as the major succinylation transferase).

A1 Glucose(+)+DMSO

A2 Glucose(+)+Etomoxir

B1 Glucose(-)+DMSO

B2 Glucose(-)+Etomoxir

HCT-116	LS174T	HCT-116	LS174T	HCT-116	LS174T

GPX4

FH

β-actin

|  | HCT-116 | | | | LS174T | | | |
| --- | --- | --- | --- | --- | --- | --- | --- | --- |
|  | A1 | A2 | B1 | B2 | A1 | A2 | B1 | B2 |
| GPX4 | 141.47 | 141.60 | 271.69 | 50.23 | 183.75 | 303.98 | 491.75 | 47.00 |
|  | 129.14 | 154.46 | 303.43 | 38.47 | 257.40 | 255.02 | 407.22 | 49.74 |
|  | 103.81 | 143.40 | 185.57 | 24.16 | 204.79 | 201.57 | 351.28 | 61.59 |
| FH | 52.66 | 43.97 | 13.26 | 58.35 | 63.64 | 64.63 | 21.06 | 68.63 |
|  | 55.86 | 58.93 | 15.39 | 74.71 | 85.03 | 86.80 | 17.85 | 141.05 |
|  | 42.96 | 79.45 | 19.76 | 55.47 | 57.19 | 48.03 | 9.83 | 75.31 |
| β-actin | 403.81 | 381.18 | 336.58 | 379.98 | 379.75 | 262.49 | 314.22 | 322.76 |
|  | 311.36 | 306.14 | 365.70 | 322.93 | 278.46 | 254.45 | 239.00 | 253.94 |
|  | 245.36 | 278.64 | 268.00 | 271.72 | 206.95 | 239.92 | 232.71 | 259.63 |

**30.Figure S4 A**

**MG132 experiment**

A cell + WT (wild type) + MG132

B cell + K68R + MG132

C cell + K80R + MG132

D cell + K68/80R + MG132

Transfect with HA-FH plasmid

Treat with MG132 (20 nM) and detect at 0h, 2h, 4h, 8h, 16h

**HCT-116**

HA	HA	HA	HA

Times(h) 0 2 4 8 16 0 2 4 8 16	0 2 4 8 16 0 2 4 8 16

A BC	D

β-actin	β-actin	β-actin	β-actin

ABCD

ABCD

ABCD

ABCD

ABCD

**LS174T**

HA	HA	HA	HA	Times(h)	0 2 4 8 16 0 2 4 8 16	0 2 4 8 16 0 2 4 8 16

ABCD

ABCD

ABCD

ABCD

ABCD

ABCD

|  |  | HA | | | | | β-actin | | | | |
| --- | --- | --- | --- | --- | --- | --- | --- | --- | --- | --- | --- |
| Times(h) |  | 0 | 2 | 4 | 8 | 16 | 0 | 2 | 4 | 8 | 16 |
| HCT-116 | A | 159.11 | 85.91 | 28.54 | 11.34 | 4.73 | 304.27 | 302.87 | 261.96 | 310.70 | 272.68 |
|  | B | 160.26 | 108.10 | 77.67 | 28.39 | 14.10 | 274.07 | 299.51 | 259.80 | 257.34 | 263.45 |
|  | C | 188.88 | 130.49 | 72.26 | 33.21 | 18.01 | 285.66 | 316.28 | 317.14 | 292.53 | 310.62 |
|  | D | 194.25 | 167.40 | 122.31 | 54.29 | 36.99 | 233.73 | 266.80 | 261.16 | 284.52 | 274.17 |
|  | A-2 | 208.85 | 71.71 | 36.91 | 13.54 | 6.00 | 249.25 | 292.79 | 271.64 | 264.76 | 238.54 |
|  | B-2 | 232.30 | 151.60 | 70.97 | 38.12 | 15.44 | 243.67 | 212.10 | 195.84 | 203.35 | 197.50 |
|  | C-2 | 285.20 | 212.39 | 96.38 | 61.05 | 19.44 | 218.10 | 193.83 | 198.15 | 228.32 | 201.90 |
|  | D-2 | 268.24 | 237.15 | 168.56 | 82.52 | 52.76 | 185.11 | 180.29 | 152.05 | 162.13 | 144.38 |
|  | A-3 | 200.86 | 62.02 | 41.38 | 20.90 | 7.27 | 278.49 | 259.69 | 332.94 | 306.14 | 257.57 |
|  | B-3 | 228.45 | 100.60 | 59.56 | 35.31 | 20.80 | 256.96 | 203.84 | 198.48 | 212.63 | 199.40 |
|  | C-3 | 160.15 | 116.12 | 57.26 | 31.42 | 17.27 | 193.05 | 229.63 | 201.81 | 207.83 | 197.35 |
|  | D-3 | 171.77 | 134.99 | 92.02 | 46.34 | 36.67 | 223.29 | 197.16 | 195.65 | 221.17 | 181.02 |
| LS174T | A | 248.80 | 68.13 | 40.75 | 16.48 | 7.07 | 246.47 | 281.70 | 267.40 | 283.48 | 250.66 |
|  | B | 276.78 | 134.03 | 61.52 | 52.45 | 21.67 | 231.72 | 236.90 | 252.28 | 229.57 | 253.69 |
|  | C | 231.88 | 93.27 | 71.20 | 36.57 | 23.08 | 314.67 | 317.95 | 323.67 | 279.58 | 243.67 |
|  | D | 270.51 | 201.28 | 97.12 | 58.47 | 43.86 | 234.00 | 219.74 | 191.97 | 240.34 | 183.27 |
|  | A-2 | 181.95 | 82.55 | 47.45 | 20.09 | 9.65 | 200.43 | 234.61 | 204.49 | 193.74 | 223.15 |
|  | B-2 | 166.43 | 139.39 | 76.05 | 36.27 | 17.20 | 194.38 | 200.54 | 175.16 | 178.09 | 155.45 |
|  | C-2 | 147.15 | 123.64 | 62.51 | 46.19 | 20.91 | 253.40 | 250.73 | 231.89 | 226.33 | 214.49 |
|  | D-2 | 176.42 | 180.18 | 87.25 | 59.87 | 40.07 | 199.95 | 195.40 | 181.30 | 180.49 | 179.46 |
|  | A-3 | 168.25 | 62.29 | 39.79 | 14.38 | 6.72 | 415.55 | 433.00 | 407.61 | 418.46 | 390.57 |
|  | B-3 | 187.43 | 144.37 | 91.72 | 31.74 | 17.07 | 390.04 | 405.91 | 343.13 | 380.83 | 378.25 |
|  | C-3 | 226.94 | 177.93 | 73.13 | 36.16 | 21.65 | 430.94 | 444.47 | 450.57 | 419.14 | 399.25 |
|  | D-3 | 269.14 | 260.52 | 135.10 | 69.29 | 54.14 | 340.85 | 386.11 | 343.10 | 297.62 | 312.50 |

**31.Figure S4 D**

After CPT1A knockdown, both wild‑type and mutant FH effectively induced ferroptosis in colorectal cancer cells under glucose starvation conditions.A Glucose(-)/FH(KD)

B Glucose(-)/FH(KD)+FH(WT)

C Glucose(-)/FH(KD)+FH(WT)+CPT1A(KD)

D Glucose(-)/FH(KD)+FH(K66R/K80R)

E Glucose(-)/FH(KD)+FH(K66R/K80R)+CPT1A(KD)

HCT-116	LS174T

IP:FH	IgG	FH	IgG	FH

IB:Succ

IB: CPT1A

Input:FTH1

Input:GPX4

Input: CPT1A

Input:FH

Input:β-actin

| HCT-116 | A | B | C | D | E | A | B | C | D | E |
| --- | --- | --- | --- | --- | --- | --- | --- | --- | --- | --- |
| IP:FH IB:Succ | 0.00 | 0.00 | 0.00 | 0.00 | 0.00 | 9.30 | 56.55 | 7.36 | 7.75 | 8.55 |
| IP:FH IB:CPT1A | 0.00 | 0.00 | 0.00 | 0.00 | 0.00 | 9.06 | 94.51 | 0.00 | 65.78 | 0.00 |
| Input: FTH1 |  | | | | | 140.36 | 46.77 | 26.61 | 24.90 | 19.03 |
| Input: GPX4 |  |  |  |  |  | 214.93 | 32.20 | 6.26 | 4.92 | 0.00 |
| Input:CPT1A |  |  |  |  |  | 129.57 | 141.06 | 20.81 | 135.70 | 15.72 |
| Input:FH |  |  |  |  |  | 11.57 | 31.87 | 123.93 | 148.57 | 130.48 |
| Input:β-actin |  |  |  |  |  | 419.24 | 471.80 | 397.08 | 408.90 | 389.51 |
| LS174T | A | B | C | D | E | A | B | C | D | E |
| IP:FH IB:Succ | 0.00 | 0.00 | 0.00 | 0.00 | 0.00 | 0.00 | 62.18 | 6.86 | 0.00 | 0.00 |
| IP:FH IB:CPT1A | 0.00 | 0.00 | 0.00 | 0.00 | 0.00 | 6.09 | 80.48 | 0.00 | 81.59 | 0.00 |
| Input: FTH1 |  | | | | | 155.13 | 59.33 | 30.43 | 27.73 | 33.39 |
| Input: GPX4 |  |  |  |  |  | 331.25 | 43.04 | 9.55 | 6.48 | 6.69 |
| Input:CPT1A |  |  |  |  |  | 84.52 | 96.26 | 0.00 | 73.85 | 0.00 |
| Input:FH |  |  |  |  |  | 10.24 | 69.32 | 225.80 | 243.54 | 268.12 |
| Input:β-actin |  |  |  |  |  | 315.45 | 253.89 | 262.65 | 236.37 | 299.35 |
